# Blood biomarkers of secondary outcomes following concussion: A systematic review

**DOI:** 10.3389/fneur.2023.989974

**Published:** 2023-02-28

**Authors:** Ella E. K. Swaney, Tengyi Cai, Marc L. Seal, Vera Ignjatovic

**Affiliations:** ^1^Department of Haematology, Murdoch Children's Research Institute, Melbourne, VIC, Australia; ^2^Department of Paediatrics, University of Melbourne, Melbourne, VIC, Australia; ^3^Developmental Imaging, Murdoch Children's Research Institute, Melbourne, VIC, Australia; ^4^Institute for Clinical and Translational Research, Johns Hopkins All Children's Hospital, St. Petersburg, FL, United States; ^5^Department of Pediatrics, Johns Hopkins University, Baltimore, MD, United States

**Keywords:** concussion, mild traumatic brain injury, biomarkers, secondary outcomes, systematic review

## Abstract

**Introduction:**

Blood biomarkers have been identified as an alternative tool for predicting secondary outcomes following concussion. This systematic review aimed to summarize the literature on blood biomarkers of secondary outcomes following concussion in both pediatric and adult cohorts.

**Methods:**

A literature search of Embase, Medline and PubMed was conducted. Two reviewers independently assessed retrieved studies to determine inclusion in systematic review synthesis.

**Results:**

A total of 1771 unique studies were retrieved, 58 of which were included in the final synthesis. S100B, GFAP and tau were identified as being associated with secondary outcomes following concussion. Seventeen percent of studies were performed in a solely pediatric setting.

**Conclusions:**

Validation of biomarkers associated with secondary outcomes following concussion have been largely limited by heterogeneous study cohorts and definitions of concussion and mTBI, presenting a hurdle for translation of these markers into clinical practice. Additionally, there was an underrepresentation of studies which investigated pediatric cohorts. Adult markers are not appropriate for children, therefore pediatric specific markers of secondary outcomes following concussion present the biggest gap in this field.

## 1. Introduction

Each year, ~45 million people globally will experience a concussion or mild traumatic brain injury (mTBI), which accounts for ~90% of all traumatic brain injuries (1). Whilst concussion was originally seen as a largely insignificant injury, it is now associated with long-term emotional, cognitive and physical disability (2–7).

The phenotype of a concussion can be characterized by the secondary outcomes experienced as a result of the injury, and include those that occur at an acute timepoint, as well as those experienced weeks, months or years post-injury. In this review, outcomes are defined as variables that are monitored during a study to document the impact that an exposure has on the health of a given population. The primary outcome in this study is defined as the diagnosis of concussion, with secondary outcomes being additional outcomes monitored to help interpret the results of the primary outcome (8). The specific secondary outcomes include the number, type and severity of symptoms, abnormalities observed using neuroimaging, delayed recovery post-concussion and post-concussion syndrome (PCS). Blood marker concentration and abundance can be a useful tool to measure such outcomes. Given that the secondary outcomes of a concussion define the injury itself, the ability to assess, predict and treat secondary outcomes experienced post-concussion is of high clinical importance.

There have been significant efforts to develop a tool that can be used for the prognosis of secondary outcomes post-concussion. Blood biomarkers have been identified as an alternative or complementary prognostic tool for secondary outcomes from concussion, in addition to the current protocol of clinical assessment and imaging. In 2007, S100B was introduced into the Scandinavian management guidelines for mTBI with the aim to reduce CT scans and save costs (9). More recently, the US Food and Drug Administration approved the use of glial fibrillary acidic protein (GFAP) and ubiquitin carboxyterminal hydroxylase L1 (UCH-L1) as serum biomarkers to aid in the detection of abnormal CT scans in adults with a concussion (10).

This systematic review aims:

1. To summarize the state of knowledge focusing on blood biomarkers of secondary outcomes following concussion, and
2. To analyze their potential as clinical tools for personalized treatment, with the aim of optimizing clinical care and reducing the long-term burden of concussion.

## 2. Methods

### 2.1. Study design

This systematic review was conducted based on the Preferred Reporting Items for Systematic Review and Meta-Analyses (PRISMA) guidelines (Supplementary Table 1) (11).

### 2.2. Search strategy and selection criteria

Medline, Embase and PubMed were used to identify studies based on the following criteria:

- Adult and pediatric populations who have sustained a concussion, mTBI, mild head injury or minor head injury.
- Biomarkers in blood, serum or plasma that predict or are associated with secondary outcomes following concussion.
- English language.
- Human study.
- Published between 2011 and 2021.

The specific search terms used for each database search are outlined in the Supplementary material.

The titles and abstracts of studies identified using these search terms were screened by two authors (ES and TC) to assess eligibility. Studies were excluded if they met the following criteria:

- mTBI results not presented separately from moderate or severe TBI.
- Study involves non-accidental head trauma^*^.
- Case reports, conference abstracts, comment, editorial, practice guideline, letter, meta-analyses, reviews.
- Markers indicate a feature of blood (blood pressure or oxygen saturation) instead of marker concentration.

^*^Non-accidental head trauma: head injuries that are purposefully inflicted, including those due to assault, abuse and domestic violence.

Full texts of the remaining studies were then screened by one author (ES) to confirm that they met the inclusion and exclusion criteria. Studies that passed full text review were imported into Covidence for data extraction and quality analysis.

### 2.3. Data extraction and quality analysis

Data extraction and quality analysis was conducted by one author (ES) in Covidence. The following data was extracted from included studies: title, author, year of publication, study design, sample size, age of participants, definition of concussion used, sample type, biomarkers measured, analysis method used, secondary outcomes measured, post-injury timepoint, statistical analysis methods used, key results. The Newcastle Ottawa Scale (NOS) was used to judge included studies based on sample selection, group comparativeness, and ascertainment of exposure (12). Studies received a score out of nine possible “stars” with more stars indicating a more robust study design.

## 3. Results

**Figure 1:**
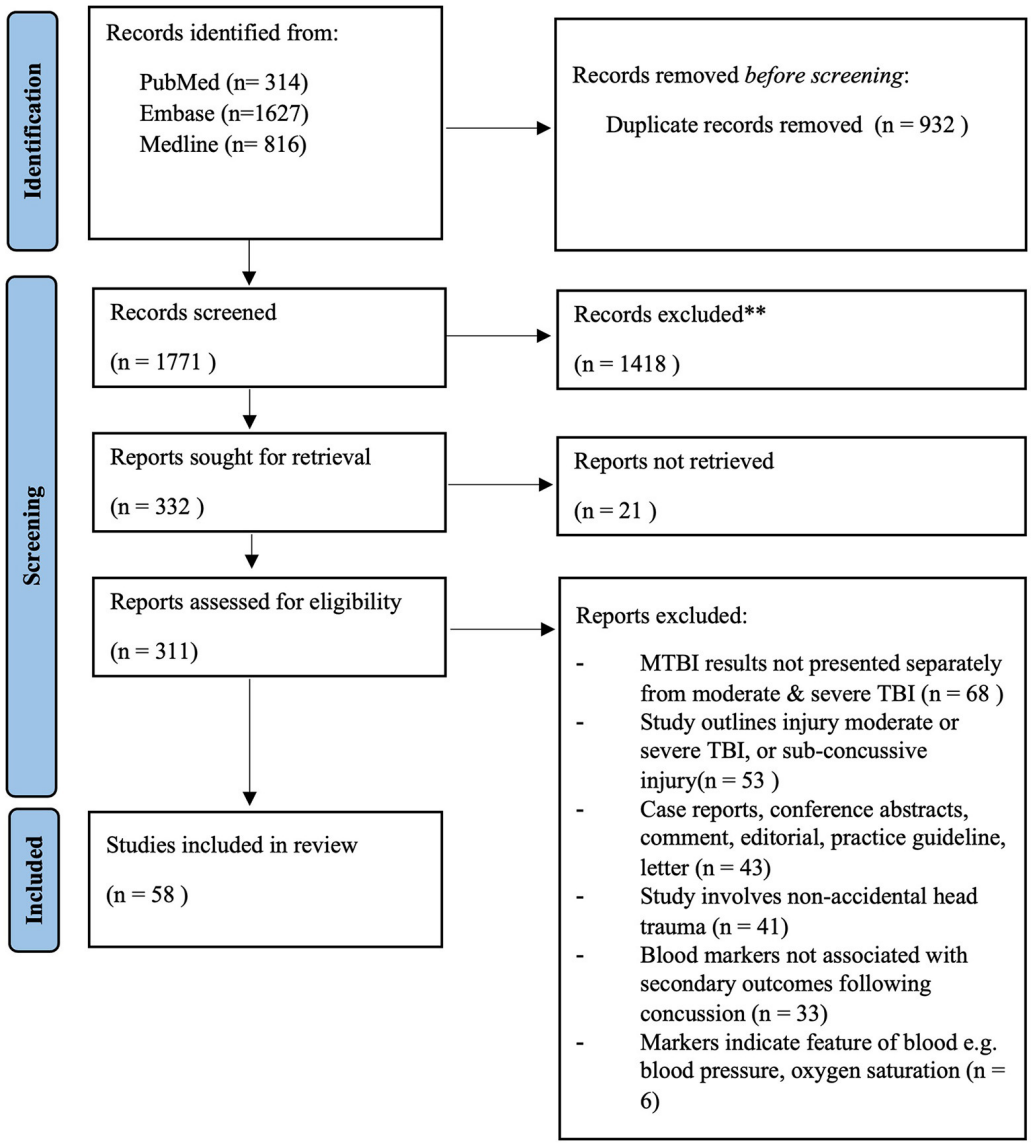
Summary of study selection process following PRISMA guidelines.

The literature search yielded 1,771 articles. Of these, 58 met the inclusion criteria (Figure 1).

NOS was used to perform quality assessment for all included studies. Separate NOS criteria was used for case-control and cross-sectional studies. The average score was 5.4 and 6.0 out of 9 for case-control and cross-sectional studies respectively (Supplementary Table 2).

### 3.1. Study characteristics

The details including patient demographics, study design, sample size, clinical outcomes and results of the 58 included studies are presented in Supplementary Table 2, with a list of clinical outcomes presented in Supplementary Table 3. Thirty studies (52%) were case-control studies, while 28 studies (48%) had a cross-sectional study design. The median participant number of all included studies was 103, ranging from 13 to 1,494, including controls. Thirty-eight studies (66%) included adult participants only, 10 studies (17%) included pediatrics participants only, and the remaining 10 studies (17%) included both adult and pediatric participants. Eighteen studies investigated biomarkers in plasma, 38 in serum and 2 in both sample types.

Of the 58 included studies, 18 studies (31%) did not include a definition of concussion or mTBI. Thirty (52%) studies included a previously developed definitions of concussion, most commonly, definitions from the Consensus Statements on Concussion in Sport (*n* = 8) and the American Congress of Rehabilitation Medicine (*n* = 4) (13–17). A summary of all included study definitions is presented in Supplementary Table 4.

### 3.2. Blood biomarkers

A total of 55 blood biomarkers were investigated across the 58 studies, with a focus on their ability to predict secondary outcomes following concussion. The biomarkers that were studied most frequently were S100B (*n* = 18), tau (*n* = 14), GFAP (*n* = 12), NFL (*n* = 8) and UCH-L1 (*n* = 5). A summary of all biomarkers investigated across included studies, and the number of times that each biomarker was investigated is presented in Table 1.

**Table 1:** Blood biomarkers and the number of studies in which they were investigated.

**Protein name**	**Acronym**	**Number of studies included**
2',3'-cyclic-nucleotide 3'-phospodiesterase	CNPase	1
3-hydroxykynurenine	3HK	2
Adrenocorticotropic hormone	ACTH	1
Alpha-synuclein	-	1
apoA-I	apoA-I	1
Beta amyloid peptide 40	AB40	1
Beta-amyloid peptide 42	AB42	3
Brain derived neurotrophic factor	BDNF	1
C-reactive protein	CRP	2
Calcitonin gene related peptide	CGRP	1
Cleaved tau protein	CTP	1
Cortisol	-	2
Dehydroepiandrosterone sulfate	DHEA-S	1
Excitatory amino acid transporter 1	EAAT1	1
Exosome enriched marker	CD81	1
Free thyroxine 4	FT-4	1
Glial fibrillary acidic protein	GFAP	12
Heart Fatty Acid Binding Protein	H-FABP	1
Interferon gamma	IFN-gamma	2
Interleukin 1 beta	IL-1beta	3
Interleukin 1 receptor antagonist	IL-1RA	1
Interleukin 10	IL-10	4
Interleukin 12	IL-12	1
Interleukin 4	IL-4	1
Interleukin-6	IL-6	4
Interleukin-8	IL-8	3
Kynurenine	KYN	2
Kynurenine metabolite A	KynA	2
Microglia-macrophage-specific protein	CD11b	1
Microtubule associated protein 2	MAP2	1
Monocyte chemoattractant protein 1	MCP-1	2
Monocyte chemoattractant protein 4	MCP-4	1
Motif chemokine ligand 2	CCL2	1
Neurofilament-light	NFL	8
Neuron specific enolase	NSE	3
Neuron-derived exosomes	NDEs	1
Oligodendrocyte myelin glycoprotein	OMG	1
Peroxiredoxin 6	PRDX-6	1
Progesterone	-	1
Prolactin	-	1
Quinolinic acid	QuinA	2
S100B	S100B	18
Soluble neural cell adhesion molecule	sNCAM	1
Soluble vascular cell adhesion molecule	sVCAM-1	1
Synaptophysin	SYP	1
Synaptosome-associated protein 25	SNAP25	1
Tau	-	14
Tau-A	-	1
Tau-C	-	1
Thyroid stimulating hormone	TSH	1
Tryptophan	TRP	2
Tumor necrosis factor alpha	TNF-alpha	2
Ubiquitin-carboxyterminal-hydrolasing enzyme	UCH-L1	5
Visinin-like protein-1	VILIP-1	1
Von Willebrand Factor	vWF	1

The different types of analysis methods used, and the number of times they were used is presented in Table 2. Fourteen different analysis methods were used across the 58 studies included in this review. Most pertinently, 41 studies utilized some type of immunoassay, including electrochemiluminescence assay, radioimmunoassay and multiplex immunoassay. Importantly, 16 of the 41 studies which utilized immunoassays used Quanterix's Single Molecule Array, a rapidly emerging digital immunoassay platform for measurement of fluid biomarkers in serum or plasma.

**Table 2:** Analysis methods and the number of studies in which they were used.

**Analysis method name**	**Acronym**	Number of studies included^*^
Electrochemiluminescence assay	-	14
Enzyme immunoassay	-	3
Enzyme linked immunosorbent assay	ELISA	11
High-performance liquid chromatography	HPLC	2
Immunoassay (unspecified)	-	4
Immunofluorescence assay	-	1
Immunonephelometric assay	-	1
Micro-RNA polymerase Chain reaction	Micro-RNA PCR	2
Microparticle based immunoassay	-	1
Multiplex immunoassay	-	6
Radioimmunoassay	-	1
Single molecular array	Simoa^®^	14

* Some studies utilized more than one analysis method.

### 3.3. Sample collection timepoints

There was no consistent definition for the acute timepoint of blood sample collection across included studies. The acute collection timepoint ranged from <3 h post-injury to <14 days post-injury. The most common acute collection timepoint was <24 h post-injury (*n* = 10), closely followed by <6 h post-injury (*n* = 9). Studies which utilized the 24-h timepoint investigated a wide range of markers, whereas those which utilized the 6-h timepoint predominantly investigated S100B (*n* = 6) and GFAP (*n* = 2).

When investigating intra-timepoint marker change, 36 studies did not discuss the half-life of blood markers being investigated at any given timepoint. Ten studies cited no statistically significant change in blood marker concentration within the acute collection window, whereas 12 studies cited a statistically significant change in blood marker concentration. There was no correlation seen between the length of the acute collection window and the determination of a statistically significant change in blood biomarker concentration at that timepoint.

### 3.4. Secondary outcomes following concussion

Twenty-seven clinical outcomes were described across the 58 studies, the most common being abnormal CT (*n* = 11), recovery duration (*n* = 8), persistent post-concussion symptoms (PCCS) (*n* = 6) and PCS (*n* = 4). Notably, an additional 4 studies described return to sport (RTP), or return to work or school (RTWS) as a secondary outcome following concussion.

**Figure 2:**
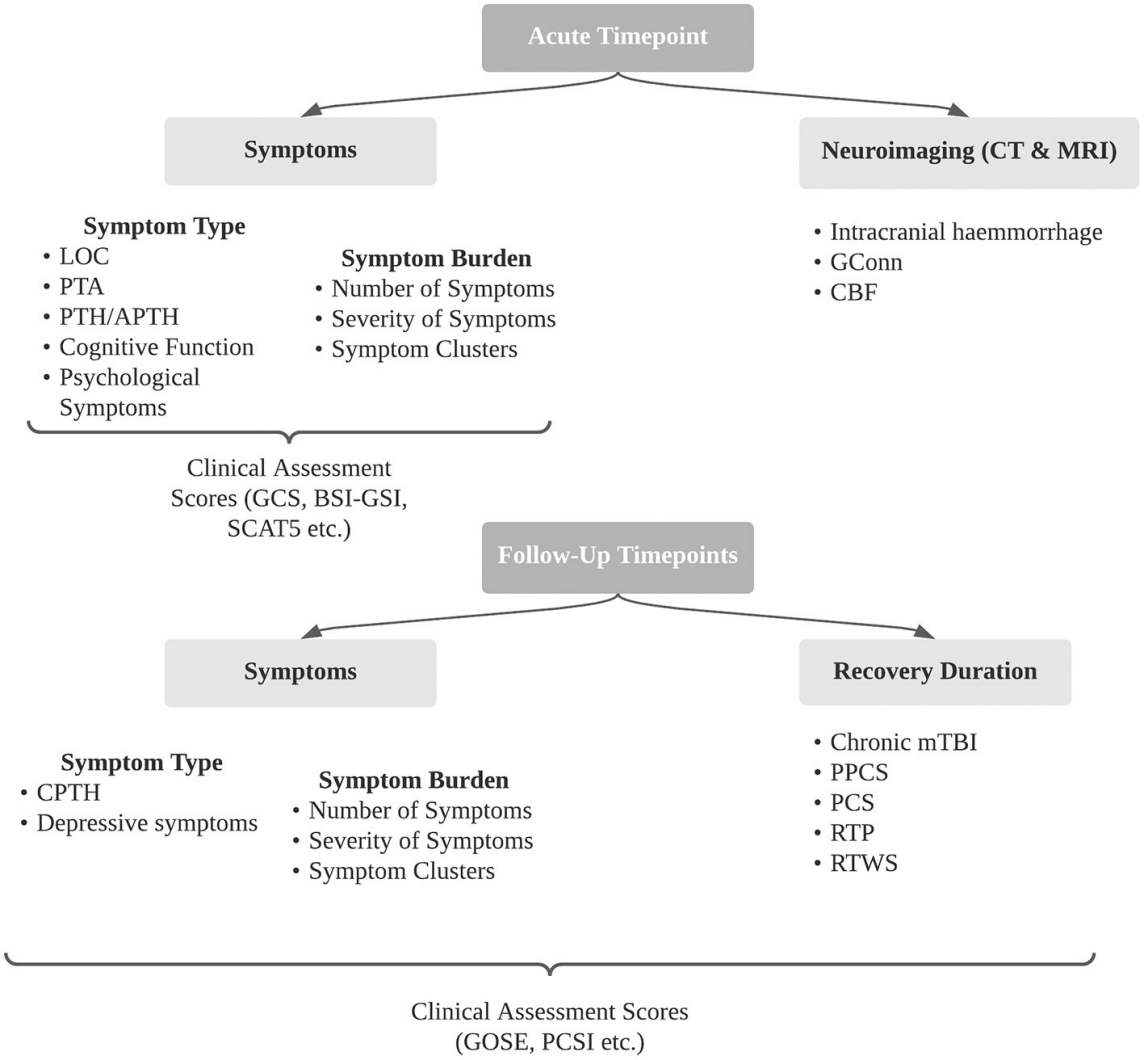
Summary of secondary outcomes following concussion identified in included studies. BSI- GSI, brief symptom inventory - Global Severity Index; CBF, cerebral blood flow; CPTH, chronic post-traumatic headache; CT, computer tomography; GConn, global connectivity; GCS, Glasgow Coma Scale; GOSE, Glasgow Outcome Scale Extended; LOC, loss of consciousness; PCS, post-concussion syndrome; PCSI, post-concussion symptoms inventory; PPCS, persistent post-concussion symptoms; PTA, post-traumatic amnesia; PTH, post-traumatic headache; RTP, return to play; RTWS, return to work and school; SCAT5, sports concussion assessment tool 5.

A summary of all secondary outcomes described in included studies is outlined in Figure 2. This review focuses on five key secondary outcomes as outlined below.

### 3.5. Abnormal CT

S100B was the most common biomarker studied in the setting of abnormal CT following concussion or mTBI. Five studies observed that S100B was able to predict intracranial pathology (*p* < 0.05), with only one of the five studies investigating pediatric patients (18–22). Conversely, 3 studies found S100B to be ineffective in predicting intracranial pathology, with two of the three studies investigating pediatric patients (23–25).

Two studies investigated the ability of the protein GFAP to detect intracranial pathology. Huebschmann et al. found GFAP to be a significant predictor of intracranial pathology in adults, whereas Forouzan et al. found it to be insignificant in predicting intracranial pathology in children and adults combined (26, 27). Diaz-Arrastia found GFAP to be unable to predict intracranial pathology, however found UCH-L1 to do so in an adult cohort (28). This was the only paper to investigate the ability of GFAP to predict intracranial pathology in plasma.

In a separate study, Forouzan et al. concluded that serum cleaved-tau protein (CTP) was able to predict intracranial pathology in pediatric and adult cohorts, whilst Stukas et al. saw no association between CT findings and serum total tau in pediatric patients (29, 30).

### 3.6. Symptom burden

Nine studies investigated blood biomarkers of symptom burden at an acute point of care.

In 2018, Asken et al. investigated the association between symptom severity in adults directly post-concussion and the concentration of serum GFAP, tau, UCH-L1, microtubule associated protein 2 (MAP2), 2',3'-cyclic-nucleotide 3'-phospodiesterase (CNPase), S100B and amyloid beta peptide 42 (AB42) and found no association between blood biomarker concentration and symptom severity (31). In 2020, McCrea et al. found no association between serum UCH-L1, tau and symptom severity directly post-injury in adult collegiate athletes, however found that athletes who experience loss of consciousness or post-traumatic amnesia have significantly higher levels of GFAP (32).

Ritchie et al. investigated the role of serum cortisol in pediatric sport-related concussion, and found low cortisol levels to be associated with symptom severity directly post-injury (33). Similarly, Begum et al. investigated 92 inflammatory markers in serum in children and adults directly post-concussion and found fibroblast growth-factor 21 (FGF21) and monocyte chemoattractant protein 1 (MCP-1) to be related with an increase in the number and severity of symptoms (34). Di Battista et al. found a between-sex difference in the relationship between symptom burden and systemic inflammation in athletes, where plasma interferon-gamma (IFN-gamma) was positively correlated with symptom severity in male athletes, but negatively correlated with symptom severity in female athletes (35). This study also observed a relationship between myeloperoxidase (MPO), tumor necrosis factor alpha (TNF-alpha) and monocyte chemoattractant protein 4 (MCP-4) and symptom severity in female athletes.

### 3.7. Recovery duration

Ten (17%) studies investigated the relationship between blood biomarkers and recovery duration. The definition of recovery duration varied between studies. Seven studies measured the number of days each participant took to completely recovery from their concussion (36–42). The remaining three studies investigated whether participants were symptom free at 30 days, 6 months and 12 months respectively (43–45).

Six studies investigated the ability of tau to predict recovery duration in both adult and pediatric cohorts. Asken et al., Anzalone et al., Hossain et al. and Kawata et al. all found an insignificant or negligible relationship between tau concentration and recovery duration (36, 37, 43, 44). Shahim et al. combined tau and S100B concentration in ice-hockey players 1 h post-concussion and found a significant correlation with the number of days for symptom resolution (38). Studies utilized both serum and plasma, however difference in samples types did not account for the inconsistency in tau concentration predicting recovery duration. Namely, Anzalone et al., Hossain et al., and Kawata et al. all investigated tau concentration in plasma, whereas Asken et al. and Shahim et al. investigated tau concentration in serum.

Three studies investigated the relationship between NFL and recovery duration in adult and pediatric cohorts and concluded that there was no significant relationship in either plasma or serum samples (36, 37, 43).

An additional 27 markers were investigated across all studies that identified recovery duration as a secondary outcome. Di Battista also investigated the evidence of a distinct peripheral inflammatory profile in sport-related concussion by studying the association between 20 plasma inflammatory biomarkers and days to recovery (39). Dehydroepiandrosterone sulfate (DHEA-S), progesterone, prolactin, MCP-1, MCP-4, interleukin-6 (IL-6) were found to be associated with recovery duration, however there was no association between recovery duration and the remaining 21 markers.

### 3.8. PPCS/PCS

Ten studies investigated blood biomarkers of PPCS and PCS. The definitions of PPCS and PCS differed between studies. Three studies defined PCS as experiencing symptoms at 3 months post-injury, whereas another study defined PCS as experiencing symptoms 1 month post-injury (23, 46–48). PPCS was measured continuously in 2 studies, where blood sample collection and cognitive testing was conducted at multiple timepoints (49, 50). In 4 other studies, PPCS was defined as continuing to experience symptoms at 48 h, 6 days, 2 weeks, and 4 months post injury (49, 51–53).

Four studies investigated S100B as a blood biomarker of PPCS or PCS. Of those four studies, three were conducted in pediatric cohorts, two of which concluded that S100B was not associated with PCS or PPCS in plasma and serum, with the remaining study concluding that patients with PCS 3 months post injury had significantly higher levels of serum S100B (23, 48, 49).

Additional biomarkers that were investigated for their ability to predict PPCS or PCS were alpha-synuclein, CRP, GFAP, interleukins, micro-ribonucleic acids (micro-RNAs), NFL, tau-A, tau-C and UCH-L1. Su et al. concluded that baseline plasma CRP levels were associated with persistent PCS in adults (46). Additionally, Shahim et al. found that serum tau-A was correlated with the duration of post-concussion symptoms (51). In a pediatric cohort, Parkin et al. found that there was a significant increase in plasma TNF-alpha protein expression at 1–4 days post-injury in children with persisting symptoms compared to those with normal recovery (49).

### 3.9. Return to play, work and school

Three studies investigated blood biomarkers of return to play (RTP) in adult athletes. All of these studies investigated tau as a biomarker for RTP, and produced conflicting results (54–56). Gill et al. and Pattinson et al. concluded that a higher concentration of plasma total tau (t-tau) was associated with longer RTP. In contrast, Mondello et al. found that there was no correlation between plasma t-tau and RTP.

One study investigated the relationship between serum S100B and return to work or school (RTWS), and found no significant association between these factors (57).

### 3.10. Statistical analysis to determine biomarker efficacy

To determine efficacy of biomarkers in predicting secondary outcomes, a number of statistical tests were used across the 58 studies, including parametric and non-parametric hypothesis tests, regressions, and receiver operating characteristic curves. Most commonly, receiver operating characteristic curves were used to determine the value of biomarkers in predicting secondary outcomes (n=23), whereas Spearman rank correlation coefficients were used to determine relationships between biomarkers and secondary outcomes (*n* = 10). Regression models were also commonly utilized, including uni- and multi-variate linear regression models (*n* = 4) and uni- and multi-variate logistic regression models (*n* = 4).

### 3.11. Assessment of risk and bias

This systematic review was conducted based on the PRISMA guidelines and assessed included studies based on the NOS scale. Titles and abstracts were screened by two authors to assess eligibility, and conflicts were resolved by a third author. Authors did not discuss their screening during the process, and exclusively used the inclusion and exclusion criteria as a guide to perform screening. Any discrepancies between authors who screened studies were resolved by a third author. Data extraction of included studies was conducted using Covidence, a software which allows for effective management and streamlining of systematic reviews.

## 4. Discussion

This review identified 55 biomarkers whose association with secondary outcomes following concussion has been investigated. Twenty-five biomarkers were shown to be associated with secondary outcomes following concussion, 18 of which were only shown to be successful in only one study. Of the remaining 7 biomarkers, S100B, tau and GFAP were shown to be associated with the same secondary outcome more than once.

By definition, a higher total number of studies utilizing serum (*n* = 38) compared to plasma (*n* = 18) resulted in a higher absolute number of studies yielding statistically significant results, as determined by the various statistical tests utilized across studies, and outlined in section 3.10. However, the difference in sample types cannot explain the fact that studies investigating the same biomarker's efficacy in predicting the same secondary outcomes yielded inconsistent results.

Most studies failed to describe the correlation between blood marker concentration and the post-injury collection time (*n* = 36). Of the studies that did investigate blood marker concentration within the post-injury collection window (*n* = 22), 12 (55%) studies found a statistically significant change in blood markers within their chosen post-injury collection timepoint. A lack of insight into the half-life of blood markers may be leading studies to select an inappropriate blood marker collection window.

### 4.1. Abnormal CT

Blood biomarkers to predict intracranial pathology on CT scans have been heavily investigated in this area of research, as they are seen as a cheap, non-invasive alternative to CT scans.

As outlined previously, S100B, GFAP and UCH-L1 have all been approved for prediction of intracranial pathology on CT scans in adult cohorts (10, 23–28). The studies investigated in this review provided contradictory evidence for their effectiveness in doing so, and particularly highlighted the ineffectiveness of S100B to predict intracranial pathology in pediatric cohorts (23, 24). The use of blood biomarkers to predict intracranial pathology in place of CT scans would be particularly useful in pediatric cohorts as the process to obtain a CT scan is often logistically difficult in that children often have to be anesthetized (58). As such, this review has highlighted the continued clinical need for blood biomarkers of secondary outcomes following concussion.

### 4.2. Symptom burden

Blood biomarkers of symptom severity were identified as being of high importance to improve understanding of the underlying pathogenesis of concussion (33). With such an understanding, neurometabolic pathways could be targeted to accelerate therapeutic advancement to treat concussion (34).

Despite the pathophysiology of concussion being largely unknown, 6 of the 9 studies (67%) that investigated symptom burden in this review studied biomarkers related to inflammation or hormonal disruption such as interleukins, MCPs and cortisol (33–35, 59–61). Additionally, many of these studies investigated similar inflammatory biomarkers and produced different outcomes. These findings indicate that the mechanism of concussion may be heterogeneous, with the cause of such heterogeneity not yet being attributed to particular demographic factors or injury mechanisms. As such, selecting markers that have potential pathophysiological implications may not be a robust way of detecting symptom burden following concussion.

### 4.3. Recovery duration, PPCS and PCS

Current clinical assessment tools used to diagnose concussion at an acute point of care are not effective in predicting delayed recovery from concussion, and as such clinicians are currently unable to provide targeted treatment based on a patient's predicted recovery time (62). Increasing interest has been given toward blood biomarkers of recovery duration as they would provide clinicians with a method to identify at-risk patients early, and provide personalized treatment.

Studies included heterogeneous definitions of PPCS and PCS when they identified as secondary outcomes following concussion presented heterogeneous study definitions of concussion, PPCS and PCS. Most notably, clinical parameters used to define PPCS or PCS were heterogeneous across studies. The Rivermead Post-Concussion Symptom Questionnaire was used to predict PPCS and PCS at various timepoints in 4 studies, whereas the remaining studies used different iterations of neuropsychological and cognitive performance assessments, including the Beck depression and anxiety inventory, diagnostic and statistical manual of mental disorders and the Montreal cognitive assessment (46, 51, 53).

Additionally, studies presented various reasons for why specific time periods post-concussion were used to measure recovery duration. Shahim et al. posited that the continuous monitoring of biomarkers over months and years post-injury is effective in assessing the risk of a patient developing traumatic axonal injury, which is a potential cause of long term disability (50). Conversely, Parkin et al. aimed to predict delayed recovery from concussion at 1–4 days, 1 week and 2 weeks post-concussion in order to facilitate personalized treatment as early as possible (49).

### 4.4. Return to play, work and school

A blood biomarker of return to school, work or sport was identified as being clinically useful to ensure that patient do not return to normal activity prior to full neuronal recovery (56). In particular, athletes who return to sport prematurely are at risk of long-term symptoms and deficits if they sustain a concussion, and are at higher risk of developing chronic traumatic encephalopathy (CTE) (63).

Of the four studies that investigated blood biomarkers of RTP & RTWS, there was a significant association seen between tau and days to return to sport in two studies (55, 56). This association can be attributed to not only the brain injury itself, but also to physical exertion, which increases neuronal activity, blood brain barrier permeability, neurogenesis and neuronal plasticity (64). Further study should be undertaken to better understand the influence of physical exertion on concentrations of tau in a concussion setting to indicate the robustness of tau in predicting time to RTP in athletes.

### 4.5. Adult vs. pediatric cohorts

Studies that investigated blood biomarkers of secondary outcomes of concussion in pediatric cohorts are underrepresented in this review. Additionally, unlike in adults, there is currently no blood biomarkers which are approved in a clinical setting to predict or assess any secondary outcome in children following their concussion (9, 10). Ten studies investigated solely pediatric cohorts, and an additional 10 investigated pediatric and adult cohorts together. The proportion of studies that focus on pediatric concussion does not reflect the frequency of occurrence nor the burden of concussion on children. Pediatric concussion has increased by 500% in the past 10 years, with 30–50% of children experiencing delayed recovery, and younger age being a risk factor for slower recovery (65). As such, the underrepresentation of pediatric studies in this area cannot be justified.

### 4.6. Future directions

This review has identified three key points as future considerations when investigating blood biomarkers of secondary outcomes of concussion:

- Studies should endeavor to use a previously developed and validated definition of concussion, which will allow for the effectiveness of blood biomarkers in predicting secondary outcomes to be determined more efficiently.
- Future studies should focus on untargeted analysis approaches to identify proteins that are able to differentiate between patient outcomes following concussion, which would identify more robust biomarkers that are influenced by the varying pathophysiological mechanisms of concussion to a lesser extent.
- Further study of blood biomarkers of secondary outcomes of concussion in pediatric cohorts should be undertaken. Children have the potential to experience a longer and more detrimental period of disability relative to adults following their concussion (65). Additionally, blood biomarkers which are deemed effective in predicting secondary outcomes of concussion in adults are yet to be proven effective, or are ineffective in children.

### 4.7. Conclusion

There is evidence of S100B and GFAP being effective biomarkers of intracranial pathology on CT scans, and tau as a potential biomarker for recovery duration, in adults. However, this systematic review demonstrates that whilst there have been significant efforts to identify clinically useful blood biomarkers of secondary outcomes following concussion with some success, progress has been limited by heterogeneous study cohorts and unstandardized definitions of concussion and mTBI. Additionally, there was an underrepresentation of studies which focused on pediatric cohorts and on developing clinically useful biomarkers in a pediatric setting. With the knowledge that the composition of pediatric blood differs vastly from adults, pediatric specific markers of secondary outcomes following concussion present the biggest gap in this area of research.

## Data availability statement

The original contributions presented in the study are included in the article/Supplementary material, further inquiries can be directed to the corresponding author.

## Author contributions

ES and VI conceptualized the methodology. ES and TC performed text screening. VI and MS reviewed and edited the manuscript. All authors contributed to the article and approved the submitted version.

## References

[1] Maas AIR Menon DK Ercole A McFadyen C Newcombe V David Adelson PD . Traumatic brain injury: integrated approaches to improve prevention, clinical care, and research. Lancet Neurol. (2017) 16:987–1048. 10.1016/S1474-4422(17)30371-X 29122524

[2] Zemek RL Farion KJ Sampson M McGahern C. Prognosticators of persistent symptoms following pediatric concussion: a systematic review. JAMA Pediatr. (2013) 167:259. 10.1001/2013.jamapediatrics.216 23303474

[3] Fineblit S Selci E Loewen H Ellis M Russell K. Health-related quality of life after pediatric mild traumatic brain injury/concussion: a systematic review. J Neurotrauma. (2016) 33:1561–8. 10.1089/neu.2015.4292 26916876

[4] Moran LM Taylor HG Rusin J Bangert B Dietrich A Nuss KE . Quality of life in pediatric mild traumatic brain injury and its relationship to postconcussive symptoms. J Pediatr Psychol. (2012) 37:736–44. 10.1093/jpepsy/jsr087 21994421 PMC3404451

[5] Gornall A Takagi M Clarke C Babl FE Davis GA Dunne K . Behavioral and emotional difficulties after pediatric concussion. J Neurotrauma. (2020) 37:163–9. 10.1089/neu.2018.6235 31072265

[6] Novak Z Aglipay M Barrowman N Yeates KO Beauchamp MH Gravel J . Association of persistent postconcussion symptoms with pediatric quality of life. JAMA Pediatr. (2016) 170:e162900. 10.1001/jamapediatrics.2016.2900 27775762

[7] Anderson V Rausa VC Anderson N Parkin G Clarke C Davies K . Protocol for a randomised clinical trial of multimodal postconcussion symptom treatment and recovery: the concussion essentials study. BMJ Open. (2021) 11:e041458. 10.1136/bmjopen-2020-041458 33574145 PMC7880104

[8] Ferreira JC Patino CM. Types of outcomes in clinical research. J bras pneumol. (2017) 43:5–5. 10.1590/s1806-37562017000000021 28380183 PMC5790671

[9] Calcagnile O Anell A Unden J. The addition of S100B to guidelines for management of mild head injury is potentially cost saving. BMC Neurol. (2016) 16:200. 10.1186/s12883-016-0723-z 27765016 PMC5073952

[10] Commissioner O of the. FDA Authorizes Marketing of First Blood Test to Aid in the Evaluation of Concussion in Adults. FDA. (2020). Available online at: http://www.fda.gov/news-events/press-announcements/fda-authorizes-marketing-first-blood-test-aid-evaluation-concussion-adults (accessed April 1, 2020).

[11] Page MJ McKenzie JE Bossuyt PM Boutron I Hoffmann TC Mulrow CD . The PRISMA 2020 statement: an updated guideline for reporting systematic reviews. BMJ. (2021) 372:n71. 10.1136/bmj.n71 33782057 PMC8005924

[12] Ottawa Hospital Research Institute. Available online at: http://www.ohri.ca/programs/clinical_epidemiology/oxford.asp (accessed November 18, 2021).

[13] Aubry M Cantu R Dvorak J Graf-Baumann T Johnston K Kelly J . Summary and agreement statement of the First International Conference on Concussion in Sport, Vienna 2001. Recommendations for the improvement of safety and health of athletes who may suffer concussive injuries. Br J Sports Med. (2002) 36:6–10. 10.1136/bjsm.36.1.6 11867482 PMC1724447

[14] McCrory P Meeuwisse W Dvorak J Aubry M Bailes J Broglio S . Consensus statement on concussion in sport—the 5 ^th^ international conference on concussion in sport held in Berlin, October 2016. Br J Sports Med. (2017) 51. 10.1136/bjsports-2017-097699 28446457

[15] McCrory P Meeuwisse WH Aubry M Cantu B Dvorák J Echemendia RJ . Consensus statement on concussion in sport: the 4th International Conference on Concussion in Sport held in Zurich, November 2012. Br J Sports Med. (2013) 47:250–8. 10.1136/bjsports-2013-092313 23479479

[16] McCrory P Meeuwisse W Johnston K Dvorak J Aubry M Molloy M . consensus statement on concussion in sport: the 3rd international conference on concussion in sport held in Zurich, November 2008. J Athl Train. (2009) 44:434–48. 10.4085/1062-6050-44.4.434 19593427 PMC2707064

[17] Silverberg ND Gardner AJ Brubacher JR Panenka WJ Li JJ Iverson GL. Systematic review of multivariable prognostic models for mild traumatic brain injury. J Neurotrauma. (2015) 32:517–26. 10.1089/neu.2014.3600 25222514

[18] Simon-Pimmel J Lorton F Guiziou N Levieux K Vrignaud B Masson D . Serum S100beta neuroprotein reduces use of cranial computed tomography in children after minor head trauma. Shock. (2015) 44:410–6. 10.1097/SHK.0000000000000442 26196846

[19] Asadollahi S Heidari K Taghizadeh M Seidabadi AM Jamshidian M Vafaee A . Reducing head computed tomography after mild traumatic brain injury: screening value of clinical findings and S100B protein levels. Brain Inj. (2016) 30:172–8. 10.3109/02699052.2015.1091504 26671496

[20] Kahouadji S Salamin P Praz L Coiffier J Frochaux V Durif J . S100B blood level determination for early management of ski-related mild traumatic brain injury: a pilot study. Front Neurol. (2020) 11:856. 10.3389/fneur.2020.00856 32922357 PMC7456809

[21] Oris C Bouillon-Minois JB Pinguet J Kahouadji S Durif J Mesle V . Predictive performance of blood S100B in the management of patients over 65 years old with mild traumatic brain injury. J Gerontol A Biol Sci Med Sci. (2021) 76:1471–9. 10.1093/gerona/glab055 33647933

[22] Haselmann V Schamberger C Trifonova F Ast V Froelich MF Straus M . Plasma-based S100B testing for management of traumatic brain injury in emergency setting. Pract Lab Med. (2021) 26:e00236. 10.1016/j.plabm.2021.e00236 34041343 PMC8141926

[23] Babcock L Byczkowski T Wade SL Ho M Bazarian JJ. Inability of S100B to predict postconcussion syndrome in children who present to the emergency department with mild traumatic brain injury: a brief report. Pediatr Emerg Care. (2013) 29:458–61. 10.1097/PEC.0b013e31828a202d 23528506 PMC3623559

[24] Bazarian JJ Blyth BJ He H Mookerjee S Jones C Kiechle K . Classification accuracy of serum Apo A-I and S100B for the diagnosis of mild traumatic brain injury and prediction of abnormal initial head computed tomography scan. J Neurotrauma. (2013) 30:1747–54. 10.1089/neu.2013.2853 23758329 PMC4047844

[25] Czeiter E Amrein K Gravesteijn BY Lecky F Menon DK Mondello S . Blood biomarkers on admission in acute traumatic brain injury: relations to severity, CT findings and care path in the CENTER-TBI study. EBioMedicine. (2020) 56:102785. 10.1016/j.ebiom.2020.102785 32464528 PMC7251365

[26] Huebschmann NA Luoto TM Karr JE Berghem K Blennow K Zetterberg H . Comparing Glial Fibrillary Acidic Protein (GFAP) in serum and plasma following mild traumatic brain injury in older adults. Front Neurol. (2020) 11:1054. 10.3389/fneur.2020.01054 33071938 PMC7530818

[27] Forouzan A Barzegari H Hosseini O Delirrooyfard A. The diagnostic competence of glial fibrillary acidic protein in mild traumatic brain injury and its prognostic value in patient recovery. Turk Neurosurg. (2021) 31:355–60. 10.5137/1019-5149.JTN.31021-20.2 33978198

[28] Diaz-Arrastia R Wang KKW Papa L Sorani MD Yue JK Puccio AM . Acute biomarkers of traumatic brain injury: relationship between plasma levels of ubiquitin C-terminal hydrolase-L1 and glial fibrillary acidic protein. J Neurotrauma. (2014) 31:19–25. 10.1089/neu.2013.3040 23865516 PMC3880090

[29] Forouzan A Motamed H Delirrooyfard A Zallaghi S. Serum cleaved tau protein and clinical outcome in patients with minor head trauma. Open Access Emerg med. (2020) 12:7–12. 10.2147/OAEM.S217424 32021498 PMC6980837

[30] Stukas S Gill J Wellington C Higgins V Adeli K Frndova H . Characterisation of serum total tau following paediatric traumatic brain injury: a case-control study. Lancet Child Adolesc Health. (2019) 3:558–67. 10.1016/S2352-4642(19)30194-4 31231066

[31] Asken BM Bauer RM DeKosky ST Houck ZM Moreno CC Jaffee MS . Concussion BASICS II: baseline serum biomarkers, head impact exposure, and clinical measures. Neurology. (2018) 91:e2123–32. 10.1212/WNL.0000000000006616 30404782 PMC6282234

[32] McCrea M Broglio SP McAllister TW Gill J Giza CC Huber DL . Association of blood biomarkers with acute sport-related concussion in collegiate athletes: findings from the NCAA and department of defense CARE consortium. JAMA Netw Open. (2020) 3:e1919771. 10.1001/jamanetworkopen.2019.19771 31977061 PMC6991302

[33] Ritchie EV Emery C Debert CT. Analysis of serum cortisol to predict recovery in paediatric sport-related concussion. Brain Inj. (2018) 32:523–8. 10.1080/02699052.2018.1429662 29400570

[34] Begum G Reddy R Yakoub KM Belli A Davies DJ Di Pietro V. Differential expression of circulating inflammatory proteins following sport-related traumatic brain injury. Int J Mol Sci. (2020) 21:1216. 10.3390/ijms21041216 32059364 PMC7072845

[35] Di Battista AP Churchill N Rhind SG Richards D Hutchison MG. The relationship between symptom burden and systemic inflammation differs between male and female athletes following concussion. BMC Immunol. (2020) 21:11. 10.1186/s12865-020-0339-3 32164571 PMC7068899

[36] Asken BM Yang Z Xu H Weber AG Hayes RL Bauer RM . Acute effects of sport-related concussion on serum glial fibrillary acidic protein, ubiquitin C-terminal hydrolase L1, Total Tau, and neurofilament light measured by a multiplex assay. J Neurotrauma. (2020) 37:1537–45. 10.1089/neu.2019.6831 32024456

[37] Kawata K Mitsuhashi M Aldret R. A preliminary report on brain-derived extracellular vesicle as novel blood biomarkers for sport-related concussions. Front Neurol. (2018) 9:239. 10.3389/fneur.2018.00239 29706930 PMC5906531

[38] Shahim P Tegner Y Wilson DH Randall J Skillback T Pazooki D . Blood biomarkers for brain injury in concussed professional ice hockey players. JAMA Neurol. (2014) 71:684–92. 10.1001/jamaneurol.2014.367 24627036

[39] Di Battista AP Churchill N Rhind SG Richards D Hutchison MG. Evidence of a distinct peripheral inflammatory profile in sport-related concussion. J Neuroinflammation. (2019) 16:17. 10.1186/s12974-019-1402-y 30684956 PMC6347801

[40] Di Battista AP Rhind SG Churchill N Richards D Lawrence DW Hutchison MG. Peripheral blood neuroendocrine hormones are associated with clinical indices of sport-related concussion. Sci Rep. (2019) 9:18605. 10.1038/s41598-019-54923-3 31819094 PMC6901546

[41] Shahim P Mattsson N Macy EM Crimmins DL Ladenson JH Zetterberg H . Serum visinin-like protein-1 in concussed professional ice hockey players. Brain Inj. (2015) 29:872–6. 10.3109/02699052.2015.1018324 25955117

[42] Nitta ME Savitz J Nelson LD Teague TK Hoelzle JB McCrea MA . Acute elevation of serum inflammatory markers predicts symptom recovery after concussion. Neurology. (2019) 93:e497–507. 10.1212/WNL.0000000000007864 31270219 PMC6693429

[43] Anzalone AJ Turner SM Baleztena AC McGuffin T Creed K Jeromin A . Blood biomarkers of sports-related concussion in pediatric athletes. Clin J Sport Med. (2021) 31:250–6. 10.1097/JSM.0000000000000735 30839351

[44] Hossain I Mohammadian M Takala RSK Tenovuo O Azurmendi Gil L Frantzen J . admission levels of total tau and beta-amyloid isoforms 1-40 and 1-42 in predicting the outcome of mild traumatic brain injury. Front Neurol. (2020) 11:325. 10.3389/fneur.2020.00325 32477238 PMC7237639

[45] Castello LM Salmi L Zanotti I Gardino CA Baldrighi M Settanni F . The increase in copeptin levels in mild head trauma does not predict the severity and the outcome of brain damage. Biomark med. (2018) 12:555–63. 10.2217/bmm-2018-0041 29620422

[46] Su SH Xu W Li M Zhang L Wu YF Yu F . Elevated C-reactive protein levels may be a predictor of persistent unfavourable symptoms in patients with mild traumatic brain injury: a preliminary study. Brain Behav Immun. (2014) 38:111–7. 10.1016/j.bbi.2014.01.009 24456846

[47] Mitra B Rau TF Surendran N Brennan JH Thaveenthiran P Sorich E . Plasma micro-RNA biomarkers for diagnosis and prognosis after traumatic brain injury: a pilot study. J Clin Neurosci. (2017) 38:37–42. 10.1016/j.jocn.2016.12.009 28117263

[48] Kelmendi FM Morina AA Mekaj AY Dragusha S Ahmeti F Alimehmeti R . Ability of S100B to predict post-concussion syndrome in paediatric patients who present to the emergency department with mild traumatic brain injury. Br J Neurosurg. (2021) 1–6. 10.1080/02688697.2021.1878487 33565911

[49] Parkin GM Clarke C Takagi M Hearps S Babl FE Davis GA . Plasma tumor necrosis factor alpha is a predictor of persisting symptoms post-concussion in children. J Neurotrauma. (2019) 36:1768–75. 10.1089/neu.2018.6042 30569819

[50] Shahim P Politis A van der Merwe A Moore B Chou YY Pham DL . Neurofilament light as a biomarker in traumatic brain injury. Neurology. (2020) 95:e610–22. 10.1212/WNL.0000000000009983 32641538 PMC7455357

[51] Shahim P Linemann T Inekci D Karsdal MA Blennow K Tegner Y . Serum tau fragments predict return to play in concussed professional ice hockey players. J Neurotrauma. (2016) 33:1995–9. 10.1089/neu.2014.3741 25621407

[52] Ye L Zhang D Shao M Zhao P Yin B Zhuang J . Lower Posttraumatic alpha-synuclein level associated with altered default mode network connectivity following acute mild traumatic brain injury. Front Neural Circuits. (2019) 13:26. 10.3389/fncir.2019.00026 31040769 PMC6476917

[53] Studer M Goeggel Simonetti B Heinks T Steinlin M Leichtle A Berger S . Acute S100B in serum is associated with cognitive symptoms and memory performance 4 months after paediatric mild traumatic brain injury. Brain Inj. (2015) 29:1667–73. 10.3109/02699052.2015.1075250 26502808

[54] Mondello S Guedes VA Lai C Jeromin A Bazarian JJ Gill JM. Sex differences in circulating T-Tau trajectories after sports-concussion and correlation with outcome. Front Neurol. (2020) 11:651. 10.3389/fneur.2020.00651 32733367 PMC7358531

[55] Gill J Livingston W Merchant-Borna K Bazarian J Jeromin A. Acute plasma tau relates to prolonged return to play after concussion. Neurology. (2017) 88:595–602. 10.1212/WNL.0000000000003587 28062722 PMC5304458

[56] Pattinson CL Meier TB Guedes VA Lai C Devoto C Haight T . Plasma biomarker concentrations associated with return to sport following sport-related concussion in collegiate athletes-A concussion assessment, research, and education (CARE) Consortium Study. JAMA netw open. (2020) 3:e2013191. 10.1001/jamanetworkopen.2020.13191 32852552 PMC7453307

[57] Ryb GE Dischinger PC Auman KM Kufera JA Cooper CC Mackenzie CF . S-100beta does not predict outcome after mild traumatic brain injury. Brain Inj. (2014) 28:1430–5. 10.3109/02699052.2014.919525 24911665

[58] Mannix R Bazarian JJ. Managing pediatric concussion in the emergency department. Ann Emerg Med. (2020) 75:762–6. 10.1016/j.annemergmed.2019.12.025 32081385

[59] Samatra DPGP Pratiwi NMD Widyadharma IPE. High IL-1beta serum as a predictor of decreased cognitive function in mild traumatic brain injury patients. Open Access Maced J Med Sci. (2018) 6:1674–7. 10.3889/oamjms.2018.390 30337986 PMC6182539

[60] Liu H Sun Y Wang Y Niu X Bai L Wang S . Mild traumatic brain injury is associated with effect of inflammation on structural changes of default mode network in those developing chronic pain. J Headache Pain. (2020) 21:135. 10.1186/s10194-020-01201-7 33228537 PMC7684719

[61] Meier TB Nitta ME Teague TK Nelson LD McCrea MA Savitz J. Prospective study of the effects of sport-related concussion on serum kynurenine pathway metabolites. Brain Behav Immun. (2020) 87:715–24. 10.1016/j.bbi.2020.03.002 32147388 PMC7316609

[62] Bressan S Clarke CJ Anderson V Takagi M Hearps SJC Rausa V . Use of the sport concussion assessment tools in the emergency department to predict persistent post-concussive symptoms in children. J Paediatr Child Health. (2020) 56:1249–56. 10.1111/jpc.14910 32436608

[63] McKee AC. The neuropathology of chronic traumatic encephalopathy: the status of the literature. Semin Neurol. (2020) 40:359–69. 10.1055/s-0040-1713632 32712946

[64] Knaepen K Goekint M Heyman EM Meeusen R. Neuroplasticity - exercise-induced response of peripheral brain-derived neurotrophic factor: a systematic review of experimental studies in human subjects. Sports Med. (2010) 40:765–801. 10.2165/11534530-000000000-00000 20726622

[65] Giza CC Kutcher JS Ashwal S Barth J Getchius TSD Gioia GA . Summary of evidence-based guideline update: evaluation and management of concussion in sports: report of the guideline development subcommittee of the American Academy of Neurology. Neurology. (2013) 80:2250–7. 10.1212/WNL.0b013e31828d57dd 23508730 PMC3721093

