# Supplementary material for: Blood biomarkers of secondary outcomes following concussion: A systematic review
Source: Front Neurol. 2023 Feb 28;14:989974. doi: 10.3389/fneur.2023.989974 (PMC10011122; doi:10.3389/fneur.2023.989974)
Supplement: Supplementary file 1 [file Data_Sheet_1.docx]

Supplementary Material

# Supplementary Data

## Search Strategy

**Medline**

1. *brain injuries/ or *brain injuries, traumatic/ or *brain concussion/

2. *head injuries, closed/ or *post-concussion syndrome/

3. ((TBI or head-injur* or brain-injur* or head-trauma) and (mild or minor)).tw,kf.

4. concuss*.tw,kf.

5. 1 or 2 or 3 or 4

6. *Biomarkers/bl [Blood]

7. *ubiquitin/bl or *tau/ or *fatty-acid-binding-proteins/bl or *microRNAs/ or *myelin basic protein/bl or *interleukins/bl or *ghrelin/bl or *matrix metalloproteinase 9/ or *chemokine CCL11/bl

8. *glial fibrillary acidic protein/bl or *neurofilament proteins/bl

9. *S100 Calcium Binding Protein beta Subunit/bl

10. (glial-fibrillary-acidic-protein or tumor-necrosis-factor or s100).tw,kf.

11. ((blood or serum or plasma) and (biomarker* or marker*)).tw,kf.

12. 6 or 8 or 9 or 10 or 11

13. "Predictive Value of Tests"/

14. *prognosis/ or *treatment outcome/

15. (predict* or detect* or associat* or outcome*).tw,kf.

16. 13 or 14 or 15

17. 5 and 12 and 16

18. (exp animals/ or (rat or rats or mouse or mice or rodent* or swine or porcine or murine or sheep or lamb or lambs or pig or pigs or piglet or piglets or rabbit or rabbits or cat or cats or dog or dogs or cattle or bovine or monkey or monkeys or trout or marmoset or marmosets).ti.) not human*.sh.

19. limit 17 to (case reports or comment or editorial or guideline or letter or practice guideline)

20. 17 not (18 or 19)

21. limit 20 to yr="2011 -Current"

22. limit 21 to english

**Embase**

1. *brain injury/ or *head injury/ or *brain concussion/ or *postconcussion syndrome/ or exp *traumatic brain injury/

2. ((TBI or head-injur* or brain-injur* or head-trauma) and (mild or minor)).tw,kw,dq.

3. concuss*.tw,kw,dq.

4. 1 or 2 or 3

5. biological marker/

6. (glial-fibrillary-acidic-protein or tumor-necrosis-factor or s100 or ubiquitin or tau or heart-fatty-acid-binding-protein or neuron-specific-enolase or micro-RNAs or myelin-basic-protein or interleukin* or ghrelin or matrix-metallopeptidase or C-C-motif-chemokine or neurofilament).tw,kw,dq.

7. ((blood or serum or plasma) and (biomarker* or marker*)).tw,kw,dq.

8. "peptides and proteins"/ or myelin/ or neurofilament protein/ or ubiquitin/ or ubiquitin c/

9. 5 or 6 or 7 or 8

10. predictive value/

11. prognosis/

12. treatment outcome/

13. (predict* or detect* or associat* or outcome*).tw,kw,dq.

14. 10 or 11 or 12 or 13

15. 4 and 9 and 14

16. (rat or rats or mouse or mice or rodent* or swine or porcine or murine or sheep or lamb or lambs or pig or pigs or piglet or piglets or rabbit or rabbits or cat or cats or dog or dogs or cattle or bovine or monkey or monkeys or trout or marmoset or marmosets).ti. and animal experiment/

17. Animal experiment/ not (human experiment/ or human/)

18. case report/

19. limit 15 to (conference abstract or conference paper or "conference review" or editorial or letter)

20. 15 not (16 or 17 or 18 or 19)

21. limit 20 to (english language and yr="2011 -Current")

**PubMed**

Title/Abstract

“TBI" OR "head-injur*" OR "brain-injur*" OR "head-trauma"

Title/Abstract

"mild" OR "minor"

Title/Abstract

“concuss*”

(#1 AND #2) OR #3

Title/Abstract

"glial-fibrillary-acidic-protein" OR "tumor-necrosis-factor" OR "s100" OR "ubiquitin" OR "tau" OR "fatty-acid-binding-protein" OR "microRNA*" OR "myelin-basic-protein" OR "interleukins" OR "ghrelin" OR "matrix-metalloproteinase 9" OR "Chemokine-CCL11" OR "neurofilament" OR "S100-Calcium-Binding-Protein-beta-Subunit"

 Title/Abstract

"blood" OR "serum" OR "plasma”

Title/Abstract

"biomarker*" OR "marker*"

#6 AND (#5 OR #7)

 Title/Abstract

"predict*" OR "detect*" OR "associate*" OR "outcome*"

All Fields

NOTNLM OR publisher[sb] OR inprocess[sb] OR pubmednotmedline[sb] OR indatareview[sb] OR pubstatusaheadofprint

All Fields

("Animal" OR "animals" OR "rat" OR "rats" OR "mouse" OR "mice" OR "rodent*" OR "swine" OR "porcine" OR "murine" OR "sheep" OR "lamb" OR "lambs" OR "pig" OR "pigs" OR "piglet" OR "piglets" OR "rabbit" OR "rabbits" OR "cat" OR "cats" OR "dog" OR "dogs" OR "cattle" OR "bovine" OR "monkey" OR "monkeys" OR "trout" OR "marmoset" OR "marmosets" OR "fly" OR "flies" OR "yeast" OR "drosophila" OR "nematode" OR "worm" OR "worms" OR "roundworm*" OR "frog" OR "frogs" OR "xenopus" OR "zebrafish" OR "zebra-fish") NOT ("human" OR "humans" OR "patient" OR "patients" OR "newborn*" OR "baby" OR "babies" OR "neonat*" OR "infan*" OR "toddler*" OR "pre-schooler*" OR "preschooler*" OR "kindergarten" OR "boy" OR "boys" OR "girl" OR "girls" OR "child" OR "children" OR "childhood" OR "adolescen*" OR "pediatric*" OR "paediatric*" OR "youth*" OR "teen" OR "teens" OR "teenage*" OR "school-aged*" OR "school-child*" OR "school-girl*" OR "school-boy*" OR "schoolgirl*" OR "schoolboy*" OR "man" OR "men" OR "woman" OR "women" OR "adult" OR "adults" OR "middle-age*" OR "elderly")

#4 AND #8 AND #9 AND #10

#12 NOT #11

## References of all included studies

1. Anzalone AJ, Turner SM, Baleztena AC, McGuffin T, Creed K, Jeromin A, et al. Blood Biomarkers of Sports-Related Concussion in Pediatric Athletes. Clin J Sport Med. 2021 May 1;31(3):250–6.

2. Asadollahi S, Heidari K, Taghizadeh M, Seidabadi AM, Jamshidian M, Vafaee A, et al. Reducing head computed tomography after mild traumatic brain injury: Screening value of clinical findings and S100B protein levels. Brain Inj. 2016;30(2):172–8.

3. Ashina H, Al-Khazali HM, Iljazi A, Ashina S, Jorgensen NR, Amin FM, et al. Low plasma levels of calcitonin gene-related peptide in persistent post-traumatic headache attributed to mild traumatic brain injury. Cephalalgia. 2020;40(12):1276–82.

4. Asken BM, Bauer RM, DeKosky ST, Houck ZM, Moreno CC, Jaffee MS, et al. Concussion BASICS II: Baseline serum biomarkers, head impact exposure, and clinical measures. Neurology. 2018;91(23):e2123–32.

5. Asken BM, Yang Z, Xu H, Weber AG, Hayes RL, Bauer RM, et al. Acute Effects of Sport-Related Concussion on Serum Glial Fibrillary Acidic Protein, Ubiquitin C-Terminal Hydrolase L1, Total Tau, and Neurofilament Light Measured by a Multiplex Assay. J Neurotrauma. 2020;37(13):1537–45.

6. Byczkowski T, Babcock L, Mookerjee S, Bazarian JJ. Ability of S100B to predict severity and cranial CT results in children with TBI. Brain Inj. 2012;26(11):1372–80.

7. Babcock L, Byczkowski T, Wade SL, Ho M, Bazarian JJ. Inability of S100B to predict postconcussion syndrome in children who present to the emergency department with mild traumatic brain injury: a brief report. Pediatr Emerg Care. 2013;29(4):458–61.

8. Di Battista AP, Churchill N, Rhind SG, Richards D, Hutchison MG. The relationship between symptom burden and systemic inflammation differs between male and female athletes following concussion. BMC Immunol. 2020;21(1):11.

9. Bazarian JJ, Blyth BJ, He H, Mookerjee S, Jones C, Kiechle K, et al. Classification accuracy of serum Apo A-I and S100B for the diagnosis of mild traumatic brain injury and prediction of abnormal initial head computed tomography scan. J Neurotrauma. 2013;30(20):1747–54.

10. Begum G, Reddy R, Yakoub KM, Belli A, Davies DJ, Di Pietro V. Differential Expression of Circulating Inflammatory Proteins Following Sport-Related Traumatic Brain Injury. Int j mol sci. 2020;21(4).

11. Bogoslovsky T, Moore C, Gong Y, Kenney K, Diaz-Arrastia R, Wilson D, et al. Increases of plasma levels of glial fibrillary acidic protein, tau, and amyloid beta up to 90 days after traumatic brain injury. J Neurotrauma. 2017;34(1):66–73.

12. Castello LM, Salmi L, Zanotti I, Gardino CA, Baldrighi M, Settanni F, et al. The increase in copeptin levels in mild head trauma does not predict the severity and the outcome of brain damage. Biomark med. 2018;12(6):555–63.

13. Crichton AJ, Ignjatovic V, Babl F, Oakley E, Greenham M, Hearps SJC, et al. Interleukin-8 Predicts Fatigue at 12 Months Post-injury in Children with Traumatic Brain Injury. Journal of Neurotrauma [Internet]. 2019 Dec 27 [cited 2020 Oct 30]; Available from: https://www.liebertpub.com/doi/10.1089/neu.2018.6083

14. Czeiter E, Amrein K, Gravesteijn BY, Lecky F, Menon DK, Mondello S, et al. Blood biomarkers on admission in acute traumatic brain injury: Relations to severity, CT findings and care path in the CENTER-TBI study. EBioMedicine. 2020;56(101647039):102785.

15. Di Battista AP, Churchill N, Schweizer TA, Rhind SG, Richards D, Baker AJ, et al. Blood biomarkers are associated with brain function and blood flow following sport concussion. Journal of Neuroimmunology. 2018 Jun;319:1–8.

16. Di Battista AP, Rhind SG, Churchill N, Richards D, Lawrence DW, Hutchison MG. Peripheral blood neuroendocrine hormones are associated with clinical indices of sport-related concussion. Sci Rep. 2019;9(1):18605.

17. Di Battista AP, Churchill N, Rhind SG, Richards D, Hutchison MG. Evidence of a distinct peripheral inflammatory profile in sport-related concussion. J Neuroinflammation. 2019;16(1):17.

18. Diaz-Arrastia R, Wang KKW, Papa L, Sorani MD, Yue JK, Puccio AM, et al. Acute biomarkers of traumatic brain injury: relationship between plasma levels of ubiquitin C-terminal hydrolase-L1 and glial fibrillary acidic protein. Casey SS CM Cooper SR, Dams-O’Connor K, Gordon WA, Hricik AJ, Menon DK, Mukherjee P, Schnyer DM, Sinha TK, Vassar MJ, editor. J Neurotrauma. 2014;31(1):19–25.

19. Forouzan A, Motamed H, Delirrooyfard A, Zallaghi S. Serum Cleaved Tau Protein and Clinical Outcome in Patients with Minor Head Trauma. Open access emerg med. 2020;12(101570796):7–12.

20. Forouzan A, Barzegari H, Hosseini O, Delirrooyfard A. The Diagnostic Competence of Glial Fibrillary Acidic Protein in Mild Traumatic Brain Injury and Its Prognostic Value in Patient Recovery. TURK NEUROSURG. 2021;31(3):355–60.

21. Gill J, Livingston W, Merchant-Borna K, Bazarian J, Jeromin A. Acute plasma tau relates to prolonged return to play after concussion. Neurology. 2017;88(6):595–602.

22. Goetzl EJ, Elahi FM, Mustapic M, Kapogiannis D, Pryhoda M, Gilmore A, et al. Altered levels of plasma neuron-derived exosomes and their cargo proteins characterize acute and chronic mild traumatic brain injury. FASEB J. 2019;33(4):5082–8.

23. Haselmann V, Schamberger C, Trifonova F, Ast V, Froelich MF, Straus M, et al. Plasma-based S100B testing for management of traumatic brain injury in emergency setting. Pract Lab Med. 2021;26(101690848):e00236.

24. Hossain I, Mohammadian M, Takala RSK, Tenovuo O, Azurmendi Gil L, Frantzen J, et al. Admission Levels of Total Tau and beta-Amyloid Isoforms 1-40 and 1-42 in Predicting the Outcome of Mild Traumatic Brain Injury. Front Neurol. 2020;11(101546899):325.

25. Huebschmann NA, Luoto TM, Karr JE, Berghem K, Blennow K, Zetterberg H, et al. Comparing Glial Fibrillary Acidic Protein (GFAP) in Serum and Plasma Following Mild Traumatic Brain Injury in Older Adults. Front Neurol. 2020;11(101546899):1054.

26. Kahouadji S, Salamin P, Praz L, Coiffier J, Frochaux V, Durif J, et al. S100B Blood Level Determination for Early Management of Ski-Related Mild Traumatic Brain Injury: A Pilot Study. Front Neurol. 2020;11(101546899):856.

27. Kawata K, Mitsuhashi M, Aldret R. A Preliminary Report on Brain-Derived Extracellular Vesicle as Novel Blood Biomarkers for Sport-Related Concussions. Front Neurol. 2018;9(101546899):239.

28. Kelmendi FM, Morina AA, Mekaj AY, Dragusha S, Ahmeti F, Alimehmeti R, et al. Ability of S100B to predict post-concussion syndrome in paediatric patients who present to the emergency department with mild traumatic brain injury. Br J Neurosurg. 2021;(ahz, 8800054):1–6.

29. Lagerstedt L, Azurmendi L, Tenovuo O, Katila AJ, Takala RSK, Blennow K, et al. Interleukin 10 and Heart Fatty Acid-Binding Protein as Early Outcome Predictors in Patients With Traumatic Brain Injury. Front Neurol. 2020;11(101546899):376.

30. LaRocca D, Barns S, Hicks SD, Brindle A, Williams J, Uhlig R, et al. Comparison of serum and saliva miRNAs for identification and characterization of mTBI in adult mixed martial arts fighters. PLoS ONE. 2019;14(1):e0207785.

31. Liu H, Sun Y, Wang Y, Niu X, Bai L, Wang S, et al. Mild traumatic brain injury is associated with effect of inflammation on structural changes of default mode network in those developing chronic pain. J Headache Pain. 2020;21(1):135.

32. Mannix R, Eisenberg M, Berry M, Meehan WP 3rd, Hayes RL. Serum biomarkers predict acute symptom burden in children after concussion: a preliminary study. J Neurotrauma. 2014;31(11):1072–5.

33. McCrea M, Broglio SP, McAllister TW, Gill J, Giza CC, Huber DL, et al. Association of Blood Biomarkers With Acute Sport-Related Concussion in Collegiate Athletes: Findings From the NCAA and Department of Defense CARE Consortium. JAMA netw open. 2020;3(1):e1919771.

34. Meier TB, Nitta ME, Teague TK, Nelson LD, McCrea MA, Savitz J. Prospective study of the effects of sport-related concussion on serum kynurenine pathway metabolites. Brain Behav Immun. 2020;87(bbi, 8800478):715–24.

35. Meier TB, Espana L, Nitta ME, Kent Teague T, Brett BL, Nelson LD, et al. Positive association between serum quinolinic acid and functional connectivity following concussion. Brain Behav Immun. 2021;91(bbi, 8800478):531–40.

36. Mitra B, Rau TF, Surendran N, Brennan JH, Thaveenthiran P, Sorich E, et al. Plasma micro-RNA biomarkers for diagnosis and prognosis after traumatic brain injury: A pilot study. J Clin Neurosci. 2017;38(dpi, 9433352):37–42.

37. Mondello S, Guedes VA, Lai C, Jeromin A, Bazarian JJ, Gill JM. Sex Differences in Circulating T-Tau Trajectories After Sports-Concussion and Correlation With Outcome. Front Neurol. 2020;11(101546899):651.

38. Nitta ME, Savitz J, Nelson LD, Teague TK, Hoelzle JB, McCrea MA, et al. Acute elevation of serum inflammatory markers predicts symptom recovery after concussion. Neurology. 2019;93(5):e497–507.

39. Oris C, Bouillon-Minois J-B, Pinguet J, Kahouadji S, Durif J, Mesle V, et al. Predictive performance of blood S100B in the management of patients over 65 years old with mild traumatic brain injury. J Gerontol A Biol Sci Med Sci. 2021;(cba, 9502837).

40. Parkin GM, Clarke C, Takagi M, Hearps S, Babl FE, Davis GA, et al. Plasma Tumor Necrosis Factor Alpha Is a Predictor of Persisting Symptoms Post-Concussion in Children. J Neurotrauma. 2019 Jun;36(11):1768–75.

41. Pattinson CL, Meier TB, Guedes VA, Lai C, Devoto C, Haight T, et al. Plasma Biomarker Concentrations Associated With Return to Sport Following Sport-Related Concussion in Collegiate Athletes-A Concussion Assessment, Research, and Education (CARE) Consortium Study. JAMA netw open. 2020;3(8):e2013191.

42. Rhine T, Babcock L, Zhang N, Leach J, Wade SL. Are UCH-L1 and GFAP promising biomarkers for children with mild traumatic brain injury?. Brain Inj. 2016;30(10):1231–8.

43. Ritchie EV, Emery C, Debert CT. Analysis of serum cortisol to predict recovery in paediatric sport-related concussion. Brain Inj. 2018;32(4):523–8.

44. Ryb GE, Dischinger PC, Auman KM, Kufera JA, Cooper CC, Mackenzie CF, et al. S-100beta does not predict outcome after mild traumatic brain injury. Brain Inj. 2014;28(11):1430–5.

45. Samatra DPGP, Pratiwi NMD, Widyadharma IPE. High IL-1beta serum as a predictor of decreased cognitive function in mild traumatic brain injury patients. Open Access Maced J Med Sci. 2018;6(9):1674–7.

46. Shahim P, Tegner Y, Wilson DH, Randall J, Skillback T, Pazooki D, et al. Blood biomarkers for brain injury in concussed professional ice hockey players. JAMA Neurol. 2014;71(6):684–92.

47. Shahim P, Mattsson N, Macy EM, Crimmins DL, Ladenson JH, Zetterberg H, et al. Serum visinin-like protein-1 in concussed professional ice hockey players. Brain Inj. 2015;29(7–8):872–6.

48. Shahim P, Linemann T, Inekci D, Karsdal MA, Blennow K, Tegner Y, et al. Serum Tau Fragments Predict Return to Play in Concussed Professional Ice Hockey Players. J Neurotrauma. 2016;33(22):1995–9.

49. Shahim P, Tegner Y, Marklund N, Blennow K, Zetterberg H. Neurofilament light and tau as blood biomarkers for sports-related concussion. Neurology. 2018;90(20):e1780–8.

50. Shahim P, Politis A, van der Merwe A, Moore B, Chou Y-Y, Pham DL, et al. Neurofilament light as a biomarker in traumatic brain injury. Neurology. 2020;95(6):e610–22.

51. Siman R, Cui H, Wewerka SS, Hamel L, Smith DH, Zwank MD. Serum SNTF, a Surrogate Marker of Axonal Injury, Is Prognostic for Lasting Brain Dysfunction in Mild TBI Treated in the Emergency Department. Front Neurol. 2020;11(101546899):249.

52. Simon-Pimmel J, Lorton F, Guiziou N, Levieux K, Vrignaud B, Masson D, et al. Serum S100beta Neuroprotein Reduces Use of Cranial Computed Tomography in Children After Minor Head Trauma. Shock. 2015;44(5):410–6.

53. Studer M, Goeggel Simonetti B, Heinks T, Steinlin M, Leichtle A, Berger S, et al. Acute S100B in serum is associated with cognitive symptoms and memory performance 4 months after paediatric mild traumatic brain injury. Brain Inj. 2015;29(13–14):1667–73.

54. Stukas S, Gill J, Wellington C, Higgins V, Adeli K, Frndova H, et al. Characterisation of serum total tau following paediatric traumatic brain injury: a case-control study. Lancet Child Adolesc Health. 2019;3(8):558–67.

55. Su S-H, Xu W, Li M, Zhang L, Wu Y-F, Yu F, et al. Elevated C-reactive protein levels may be a predictor of persistent unfavourable symptoms in patients with mild traumatic brain injury: a preliminary study. Brain Behav Immun. 2014;38(bbi, 8800478):111–7.

56. Svingos AM, Asken BM, Bauer RM, DeKosky ST, Hromas GA, Jaffee MS, et al. Exploratory study of sport-related concussion effects on peripheral micro-RNA expression. Brain Inj. 2019;33(4):1–7.

57. Xu Z, Chen Z-P, Lv X-A, Qiu H-S, Wang J-W. Predictive value of early decreased plasma ghrelin level for three-month cognitive deterioration in patients with mild traumatic brain injury. Peptides. 2014;54((Xu, Chen) Department of Neurosurgery, First Affiliated Hospital of Zhejiang Chinese Medicine University, 54 Youdian Lane, Hangzhou 310006, China):180–5.

58. Ye L, Zhang D, Shao M, Zhao P, Yin B, Zhuang J, et al. Lower Posttraumatic alpha-Synuclein Level Associated With Altered Default Mode Network Connectivity Following Acute Mild Traumatic Brain Injury. Front Neural Circuits. 2019;13(101477940):26.

59. Overview | Head injury: assessment and early management | Guidance | NICE [Internet]. NICE; [cited 2022 Feb 23]. Available from: https://www.nice.org.uk/guidance/cg176

# Supplementary Figures and Tables

## Preferred Reporting Items for Systematic Review and Meta-Analyses (PRISMA) Guidelines

| **Section and Topic** | **Item #** | **Checklist item** | **Location where item is reported** |
| --- | --- | --- | --- |
| TITLE | | |  |
| Title | 1 | Identify the report as a systematic review. | Title |
| ABSTRACT | | |  |
| Abstract | 2 | See the PRISMA 2020 for Abstracts checklist. | Abstract |
| INTRODUCTION | | |  |
| Rationale | 3 | Describe the rationale for the review in the context of existing knowledge. | Section 1 |
| Objectives | 4 | Provide an explicit statement of the objective(s) or question(s) the review addresses. | Section 1 |
| METHODS | | |  |
| Eligibility criteria | 5 | Specify the inclusion and exclusion criteria for the review and how studies were grouped for the syntheses. | Section 2.2 |
| Information sources | 6 | Specify all databases, registers, websites, organisations, reference lists and other sources searched or consulted to identify studies. Specify the date when each source was last searched or consulted. | Section 2.2 |
| Search strategy | 7 | Present the full search strategies for all databases, registers and websites, including any filters and limits used. | Supplementary Files |
| Selection process | 8 | Specify the methods used to decide whether a study met the inclusion criteria of the review, including how many reviewers screened each record and each report retrieved, whether they worked independently, and if applicable, details of automation tools used in the process. | Section 2.3 |
| Data collection process | 9 | Specify the methods used to collect data from reports, including how many reviewers collected data from each report, whether they worked independently, any processes for obtaining or confirming data from study investigators, and if applicable, details of automation tools used in the process. | Section 2.3 |
| Data items | 10a | List and define all outcomes for which data were sought. Specify whether all results that were compatible with each outcome domain in each study were sought (e.g. for all measures, time points, analyses), and if not, the methods used to decide which results to collect. | Section 2.3 |
|  | 10b | List and define all other variables for which data were sought (e.g. participant and intervention characteristics, funding sources). Describe any assumptions made about any missing or unclear information. | Section 2.3 |
| Study risk of bias assessment | 11 | Specify the methods used to assess risk of bias in the included studies, including details of the tool(s) used, how many reviewers assessed each study and whether they worked independently, and if applicable, details of automation tools used in the process. | Section 2.3 |
| Effect measures | 12 | Specify for each outcome the effect measure(s) (e.g. risk ratio, mean difference) used in the synthesis or presentation of results. | N/A |
| Synthesis methods | 13a | Describe the processes used to decide which studies were eligible for each synthesis (e.g. tabulating the study intervention characteristics and comparing against the planned groups for each synthesis (item #5)). | N/A |
|  | 13b | Describe any methods required to prepare the data for presentation or synthesis, such as handling of missing summary statistics, or data conversions. | N/A |
|  | 13c | Describe any methods used to tabulate or visually display results of individual studies and syntheses. | N/A |
|  | 13d | Describe any methods used to synthesize results and provide a rationale for the choice(s). If meta-analysis was performed, describe the model(s), method(s) to identify the presence and extent of statistical heterogeneity, and software package(s) used. | N/A |
|  | 13e | Describe any methods used to explore possible causes of heterogeneity among study results (e.g. subgroup analysis, meta-regression). | N/A |
|  | 13f | Describe any sensitivity analyses conducted to assess robustness of the synthesized results. | N/A |
| Reporting bias assessment | 14 | Describe any methods used to assess risk of bias due to missing results in a synthesis (arising from reporting biases). | N/A |
| Certainty assessment | 15 | Describe any methods used to assess certainty (or confidence) in the body of evidence for an outcome. | N/A |
| RESULTS | | |  |
| Study selection | 16a | Describe the results of the search and selection process, from the number of records identified in the search to the number of studies included in the review, ideally using a flow diagram. | Figure 1 |
|  | 16b | Cite studies that might appear to meet the inclusion criteria, but which were excluded, and explain why they were excluded. | N/A |
| Study characteristics | 17 | Cite each included study and present its characteristics. | Supplementary Materials |
| Risk of bias in studies | 18 | Present assessments of risk of bias for each included study. | Supplementary Materials |
| Results of individual studies | 19 | For all outcomes, present, for each study: (a) summary statistics for each group (where appropriate) and (b) an effect estimate and its precision (e.g. confidence/credible interval), ideally using structured tables or plots. | Supplementary Materials |
| Results of syntheses | 20a | For each synthesis, briefly summarise the characteristics and risk of bias among contributing studies. | N/A |
|  | 20b | Present results of all statistical syntheses conducted. If meta-analysis was done, present for each the summary estimate and its precision (e.g. confidence/credible interval) and measures of statistical heterogeneity. If comparing groups, describe the direction of the effect. | N/A |
|  | 20c | Present results of all investigations of possible causes of heterogeneity among study results. | N/A |
|  | 20d | Present results of all sensitivity analyses conducted to assess the robustness of the synthesized results. | N/A |
| Reporting biases | 21 | Present assessments of risk of bias due to missing results (arising from reporting biases) for each synthesis assessed. | N/A |
| Certainty of evidence | 22 | Present assessments of certainty (or confidence) in the body of evidence for each outcome assessed. | N/A |
| DISCUSSION | | |  |
| Discussion | 23a | Provide a general interpretation of the results in the context of other evidence. | Section 4 |
|  | 23b | Discuss any limitations of the evidence included in the review. | Section 4 |
|  | 23c | Discuss any limitations of the review processes used. | Section 4 |
|  | 23d | Discuss implications of the results for practice, policy, and future research. | Section 4.5 |
| OTHER INFORMATION | | |  |
|  | | |  |
| Registration and protocol | 24a | Provide registration information for the review, including register name and registration number, or state that the review was not registered. | Not registered |
|  | 24b | Indicate where the review protocol can be accessed, or state that a protocol was not prepared. | Not prepared |
|  | 24c | Describe and explain any amendments to information provided at registration or in the protocol. | N/A |
| Support | 25 | Describe sources of financial or non-financial support for the review, and the role of the funders or sponsors in the review. | N/A |
| Competing interests | 26 | Declare any competing interests of review authors. | N/A |
| Availability of data, code and other materials | 27 | Report which of the following are publicly available and where they can be found: template data collection forms; data extracted from included studies; data used for all analyses; analytic code; any other materials used in the review. | N/A |

## Summary of Studies Included in this Systematic Review

|  | Author/ Year | Study Design | Sample Size | Age Group | Age | Blood Marker Measure | Secondary Outcome Measure | Results | NOS |
| --- | --- | --- | --- | --- | --- | --- | --- | --- | --- |
| (1) | Anzalone (2019) | Case-control | Concussed athletes: 97, Non-concussed athletes: 30 | Paediatric and Adult | Age Range: 12-23 | Tau, NFL | Prolonged recovery >28 days | No significant relationship between Nf-L and symptoms reported. Weak but significant inverse relationship between tau and number of symptoms reported. | 5 |
| (2) | Asadollahi (2016) | Cross-sectional | 158 | Adult | No age range. Mean (SD): 35.4 (15.8) | S100B | Abnormal CT | With a cut-off point of 0.115ug/L, S100B had a sensitivity and specificity of 94.9% and 35.4% to predict intracranial injury on CT scanning. | 6 |
| (3) | Ashina (2020) | Case-control | Persistent PTH individuals: 100, Controls: 100 | Adult | No age range. Mean (SD): 36.0 (11.7) | CGRP | Post-traumatic headache | No significant difference in plasma CGRP levels in healthy controls and individuals with persistent PTH. | 6 |
| (4) | Asken (2018) | Cross-sectional | 415 | Adult | No age range. Mean (SD): 19.0 (1.2) | AB42, Tau, S100B, UCH-L1, GFAP, MAP2, CNPase | Clinical assessment scores | No associations between baseline biomarker levels and self-reported symptom severity or performance of clinical tests. | 7 |
| (5) | Asken (2020) | Case-control | Concussion: 28, Controls: 82 | Adult | No age range. Concussion Mean (IQR): 19 (18-20), Controls Mean (IQR): 20 (19-20). | GFAP, Tau, NFL | Recovery duration | No associations between biomarker concentrations and clinical measurements post-SRC or recovery duration. | 6 |
| (6) | Babcock (2012) | Cross-sectional | mTBI: 94 | Paediatric | Age range: 2.1-18.9 | S100B | Abnormal CT | S100 is not able to differentiate between children with normal and abnormal CT who have a mild head trauma. | 7 |
| (7) | Babcock (2013) | Cross-sectional | 481 | Paediatric | No age range. Mean (SD): 14.0 (3.1) | S100B | PCS | No association between S100B levels and PCS for children with mTBI | 6 |
| (8) | Di Battista (2020) | Cross-sectional | 40 | Adult | Age range: 18.9-22.3 | 10 inflammatory biomarkers | Cognitive, emotional, somatic and fatigue symptoms | Symptom severity was correlated with blood concentrations of IFN-gamma in male athletes. Symptom severity was negatively correlated with IFN-gamma, TNF-alpha and positively correlated with MCP-4 in female athletes. | 6 |
| (9) | Bazarian (2013) | Case-control | MTBI: 787, Controls: 467 | Paediatric and Adult | No age range. MTBI Mean (SD): 38.2 (19.5). Control Mean (SD): 39.2 (20.2) | S100B and apoA-I | Abnormal CT | Subjects with mTBI with an abnormal initial head CT had significantly higher median S100B levels but not apoA-I. The relative increase in median S100B levels among subjects with mTBI compared with controls was much higher for adults compared to children. | 0 |
| (10) | Begum (2020) | Case-control | Single concussion: 18, Repetitively concussed: 5, Healthy Controls: 13 | Paediatric and Adult | Age range: 16-34 | 92 inflammatory markers. | Symptom Severity and Cognitive Performance | Low levels of circulating FGF21 was associated with an increase in the number of reported symptoms and severity of symptoms. Reduced serum levels of MCP-1 were also related to an increase in the number and severity of symptoms. | 6 |
| (11) | Bogoslovsky (2017) | Case-control | MTBI: 21, Controls: 19 | Adult | Age range: 29-52 | GFAP, Tau, AB42 | Symptom severity | No relationship between GFAP and tau levels at any timepoint and functional outcome 180 days post-injury. There was a correlation between AB42 levels at Day 30 and GOSE at 180 days. | 6 |
| (12) | Castello (2018) | Case-control | Cases: 105, Controls: 22 | Adult | Age range: 59-83 | Copeptin | Recovery duration | Copeptin had a poor prognostic value and was not able to predict the presence of symptoms at 30 days post-injury. | 8 |
| (13) | Crichton (2019) | Cross-sectional | mTBI: 50 | Paediatric | No age range. Mean (SD): 11 (4.4) | IL-6, IL-8, sVCAM-1, sNCAM, NSE, S100B | Fatigue | A model including IL-8 was the best serum biomarker for estimating the probability of children experiencing fatigue at 12 months post-injury. IL-8 also significantly improved predictive models of fatigue based on severity. | 6 |
| (14) | Czeiter (2020) | Cross-sectional | Mild TBI: 1494 | Adult | Age range: 30-66 | S100B, NSE, GFAP | Abnormal CT | GFAP was more effective in discriminating between CT+ and CT- mTBI patients than clinical characteristics. Combinations of markers were less effective than GFAP alone. | 5 |
| (15) | Di Battista (2018) | Case-control | Acute concussion: 16, Healthy Controls: 15, History of Concussion: 12 | Adult | Age range: 18-22 | S100B, T vWF, BDNF, PRDX-6, MCP-1, MCP-4 | Gconn & CBF | In recently concussion athletes compared to healthy athletes: decrease in t-tau and Gconn, decrease in t-tau and CBF, decrease in Gconn with elevated PRDX-6, decrease in CBF with PRDX-6 and decrease in Gconn with elevated MCP-4. | 5 |
| (16) | Di Battista (2019) | Case-control | Concussion: 26, Uninjured: 67 | Adult | Age range: 19.3-22.2 | ACTH, Cortisol, DHEA-S, Prolactin, Progesterone, FT-4, TSH | Recovery duration and symptom clusters | Negative relationship between time to recovery and DHEA-S and progesterone. Positive relationship between time to recovery and prolactin. Cognitive, somatic, fatigue and emotion symptom clusters associated with distinct neuroendocrine signatures. | 6 |
| (17) | Di Battista (2019) | Case-control | Sports related concussion: 43, Musculoskeletal injury: 30, Healthy: 102 | Adult | Age range: 18.9-22.7 | 20 inflammatory biomarkers | Recovery duration | Significant positive correlation between days to recovery and MCP-1 and MCP-4 in athletes with SRC. | 6 |
| (18) | Diaz-Arrastia (2014) | Case-control | MTBI: 171, Control: 175 | Other: Adult | No age range. Mean (SD): 37 (14) | UCH-L1 | Abnormal CT | UCH-L1 results were significantly higher in mTBI subjects with CT abnormalities compared to those without. UCH-L1 levels were poorly predictive of complete recovery, and better at predicting poor outcome. | 6 |
| (19) | Forouzan (2020) | Cross-sectional | 86 | Paediatric and Adult | Age range: 16-90 | CTP | Abnormal CT and PCS | Significant relationship between intracranial injury and positive CTP. No correlation seen between CTP and PCS. | 7 |
| (20) | Forouzan (2020) | Cross-sectional | 176 | Paediatric and Adult | Age range: 16-90 | GFAP | Abnormal CT | GFAP was unacceptable in determining abnormal CT results, with a low sensitivity and specificity. | 6 |
| (21) | Gill (2017) | Case-control | SRC Athletes: 43, Control Athletes: 37, Nonathlete Controls: 21 | Adult | No age range. Control athletes Mean (SD): 18.7(0.67). SRC Athletes Mean (SD): 20(46.5). Non-athlete controls Mean (SD): 19.2(0.98) | Tau | RTP | SRC athletes with long RTP had higher tau overall compared to SRC athletes with short RTP. | 7 |
| (22) | Goetzl (2019) | Case-control | Acute mTBI: 18, Chronic mTBI: 14, Controls: 21 | Adult | No age range. Acute mTBI Mean (SEM): 20.67 (0.33). Chronic mTBI Mean (SEM): 19.64 (0.49). Control Mean (SEM): 20.62 (0.39) | 15 plasma NDEs | Chronic mTBI | Plasma levels of NDEs were decreased significantly relative to those of controls in acute but not chronic mTBI. NDE levels of five neuropathological proteins were significantly increased in acute and chronic mTBI> | 7 |
| (23) | Haselmann (2021) | Case-control | mTBI: 20, Melanoma Controls: 46 | Adult | Age range: 18-89 | S100B | Abnormal CT | Plasma and serum S100B levels were highly correlated with CCT-negative and CCT-positive mTBI patient outcomes. | 2 |
| (24) | Hossain (2020) | Cross-sectional | 105 | Adult | No age range. Mean (SD): 47.46 (20.25) | Tau, AB40, AB42 | Recovery duration | Admission levels of all plasma biomarkers were not significantly different between patients with complete and incomplete recovery at 6-12 months post-injury. | 6 |
| (25) | Huebschmann (2020) | Cross-sectional | 121 | Adult | Age range: >/=50 | GFAP | Abnormal CT and GOSE | GFAP was higher in those with abnormal CT scans compared to those with normal head CT scans. GFAP was significantly higher in those with poor compared to good functional outcome. GFAP was not an adequate predictor of functional outcome 1 week post-injury. | 6 |
| (26) | Kahouadji (2020) | Cross-sectional | 130 | Adult | No age range. Mean (SD): 44.8 (20.4) | S100B | Abnormal CT | S100B serum levels were able to predict abnormal CT scans with a 97% sensitivity and 11% specificity. | 5 |
| (27) | Kawata (2018) | Case-control | Concussion: 2, Ice Hockey Players: 6, Healthy Controls: 6 | Adult | No age range. Concussion Mean (SD): 26.63 (1.6), Ice Hockey Mean (SD): 26.63 (1.6), Healthy Controls Mean (SD): 31.5 (15.1). | SNAP25, NFL, tau, SYP, EAAT1, GFAP, OMG, CD11b, IL8, TNFalpha, CD81 | Recovery duration | Neuronal and microglia markers increased after concussions and potentially reflect the damage in neural cell structures and metabolic crisis due to concussion. | 4 |
| (28) | Kelmendi (2021) | Cross-sectional | 60 | Paediatric | Age range: 7-16 | S100B | PCS | Patients with signs of PCS 3 months post injury had significantly higher S100B protein levels than those without signs of PCS. | 6 |
| (29) | Lagerstedt (2020) | Cross-sectional | Complete recovery: 25, Incomplete recovery: 24 | Adult | No age range. Complete recovery Mean (SD): 40.9 (20.3), Incomplete recovery Mean (SD): 47.5 (18.7) | GFAP, NFL, S100B, H-FABP, IL-10 | GOSE | All proteins higher in patients with incomplete recovery, but no significant difference. H-FABP and NFL combined reached a specificity of 40% and sensitivity of 96% in determining recovery status. | 5 |
| (30) | LaRocca (2019) | Case-control | Low risk of mTBI: 50, Moderate risk of mTBI: 41, Very Likely risk of mTBI: 50 | Adult | No age range. Mean (SD): 26.5 (5.8) | miRNAs | Clinical assessment scores | Serum proteins demonstrated little utility in predicting TBI likelihood and quantitative associations with cognitive and balance measures. | 4 |
| (31) | Liu (2020) | Case-control | mTBI: 77, Healthy: 42 | Adult | No age range. mTBI + APTH Mean (SD): 37.47 (12.6). mTBI - APTH Mean (SD): 30.4 (12.8). Healthy Mean (SD): 35.3 (11.2). | IL-1beta, IL-6, IL-12, IL-4, IL-10, CCL2, MCP-1, IL-8, IFN-gamma, TNF-alpha | APTH & CPTH | CCL2 elevated in patients with APTH. CCL2 levels persistently increased in patients developing CPTH 3 months post-injury. | 3 |
| (32) | Mannix (2014) | Cross-sectional | 13 | Paediatric and Adult | Age range: 11-21 | GFAP | Symptom burden 1 month post-injury | Initial GFAP levels associated with follow-up symptom burden up to 1 month after injury, follow-up GFAP levels did not correlate with symptom burden. | 6 |
| (33) | McCrea (2020) | Case-control | Concussion: 264, Contact Sport Controls: 138, Non-contact Sport Controls: 102 | Adult | No age range. Concussion Mean (SD): 19.08 (1.24). Contact Sport Control Mean (SD): 19.03 (1.27). Non-contact Sport Control Mean (SD): 19.39 (1.25) | GFAP, UCH-L1, Tau, NFL | LOC and PTA | Athletes with LOC or PTA had significantly higher levels of GFAP than athletes with neither LOC or PTA at the acute post-injury timepoint. | 7 |
| (34) | Meier (2020) | Case-control | Concussion: 59, Uninjured contact sport: 54, Non-contact sport: 30 | Adult | Concussion Mean (SD): 18.02 (1.58), Uninjured contact sport Mean (SD): 18.3 (1.7), Non-contact sport Mean (SD): 19.1(1.75) | TRP, KYN, KynA, 3HK, QuinA | Acute psychological symptoms | Higher KynA/3HK at the early-acute visit was significantly associated with lower BSI-GSI scores. | 6 |
| (35) | Meier (2021) | Case-control | Concussion: 37, Uninjured controls: 42 | Paediatric and Adult | No age range. SRC no prior concussion Mean (SD): 17.81 (1.83). SRC prior concussion Mean (SD): 18.05 (1.75). Control no prior concussion Mean (SD): 18.08 (1.75). Control prior concussion Mean (SD): 18.39 (1.91). | QuinA, KynA, 3HK, TRP, KYN | Gconn and depressive symptoms | There is a potential role of kynurenine pathway metabolites in altered functional connectivity following concussion. | 5 |
| (36) | Mitra (2017) | Cross-sectional | 24 | Adult | Age Range: 28.5-74 | micro-RNAs | PCS | Mir142-3p and mir423-3p potential clinical utility in differentiating between patients at greater risk of developing amnesia and therefore post-concussion syndromes. | 5 |
| (37) | Mondello (2020) | Cross-sectional | 83 | Adult | No age range. Mean (SD): 18.9 (0.97) | Tau | RTP | There was no correlation between t-tau levels and RTP equal to or more than 10 days after concussion. | 6 |
| (38) | Nitta (2019) | Case-control | Concussion: 41, Controls: 43 | Paediatric and Adult | No age range. Concussion Mean (SD): 17.8 (1.79). Control Mean (SD): 18.14 (1.67) | IL-6, IL-1beta, IL-1RA, IL-10, TNF, CRP, IFN-gamma | Recovery duration | IL-6 levels at 6 hours post-concussion were significantly associated with the duration of symptoms | 6 |
| (39) | Oris (2021) | Cross-sectional | 1172 | Adult | Age Range: 65-95 | S100B | Abnormal CT | S100B was able to detect abnormal CT (p<0.001) across 65-79, 80-89 and >/=90 years old age groups. | 8 |
| (40) | Parkin (2019) | Cross-sectional | 18 | Paediatric | Age Range: 5-18 | IL-1B, IL-6, IL-8, IL-10, S100B, tau, TNF-alpha | PPCS | There were significant increase in TNF-alpha protein expression at 1-4 days post-injury in children with persisting symptoms compared to those with normal recovery. | 6 |
| (41) | Pattinson (2020) | Cross-sectional | 127 | Adult | No age range. Mean (SD): 18.9 (1.3) | UCH-L1, tau, NFL, GFAP | RTP | A significant association between mean plasma total tau (24-48 hours post-injury) and days to return to sport (more or less than 14 days). Significantly lower concentrations of GFAP (24-48 hours post injury) in the group that took 14 days or more to return to sport. | 6 |
| (42) | Rhine (2016) | Case-control | MTBI: 25, Orthopaedic injury: 20 | Paediatric | Age range: 11-16 | GFAP, UCH-L1 | PCSS | Neither GFAP nor UCH-L1 was predictive of PCSS over the one month post injury. | 6 |
| (43) | Ritchie (2018) | Case-control | Concussion: 41, Control: 595 | Paediatric | Age range: 11-12 | Cortisol | Symptom severity | Players with abnormally low cortisol experienced more symptoms, more severe symptoms and took longer to return to sport. | 7 |
| (44) | Ryb (2014) | Cross-sectional | 150 | Adult | Age range: 18-64 | S100B | RTWS | There was no association found between S100B levels and RTWS. | 6 |
| (45) | Samatra (2018) | Cross-sectional | 70 | Paediatric and Adult | Age range: 17-35 | IL-1Beta | Cognitive function | The group with high Il-1Beta serum levels were at higher risk of suffering from cognitive impairment after TBI compared to the group with normal Il-1Beta serum levels. | 4 |
| (46) | Shahim (2014) | Case-control | Concussion: 28, Controls: 47 | Adult | Age range: 19-40 | Tau, S100B, NSE | Recovery duration | Tau and S100B concentration (1 hour post-concussion) correlated with number of days for symptom resolution. | 7 |
| (47) | Shahim (2015) | Cross-sectional | 28 | Adult | No age range. Mean age: 27.2 | VILIP-1 | Recovery duration | Serum levels of VILIP-1 1 hour post-concussion did not correlate with the number of days for the concussion symptoms to resolve. | 7 |
| (48) | Shahim (2016) | Cross-sectional | 35 | Adult | Unknown | Tau-A, Tau-C | PPCS | Tau-C was significantly higher in post-concussion samples compared with preseason. Tau-A correlated with the duration of post-concussive symptoms. | 3 |
| (49) | Shahim (2018) | Case-control | 87 | Adult | Age range: 18-35 | NFL, Tau | Symptom severity and RTP | Serum NFL concentration was related to prolonged RTP time (more or less than 10 days), and discriminated players who resigned from the game due to persistent post-concussion symptoms. | 5 |
| (50) | Shahim (2020) | Case-control | Acute concussion: 45, Post-concussion symptoms: 31, Preseason controls: 28, Nonathletic controls: 14 | Adult | Age range: 22-34 | NFL | PPCS | Cerebral spinal fluid and serum NFL distinguished players with PCS >1 year from PCS </= 1 year. | 6 |
| (51) | Siman (2020) | Case-control | Concussion: 95, Controls: 40 | Paediatric and Adult | Age range: 10-86 | SNTF | Cognitive function | Elevated serum SNTF correlated significantly with persistent impairments in cognition and sensory-motor integration, and predicted poorer recovery of cognitive stress function. | 6 |
| (52) | Simon-Pimmel (2015) | Cross-sectional | 109 | Paediatric | Age range: 4 months - 18 years | S100B | Abnormal CT | Integrating serum S100B concentration in the Paediatric Emergency Care Applied Research Network (PECARN) would decrease the use of CT by 32%. | 7 |
| (53) | Studer (2015) | Case-control | Concussion: 36, Controls: 27 | Paediatric | Age range: 6-16 | S100B | PPCS | Significant correlation between S100B and post-acute cognitive PPCS as well as S100B and verbal memory performance. | 7 |
| (54) | Stukas (2019) | Case-control | Concussion: 114, Control: 416 | Paediatric | Age range: 5-14 | Tau | Abnormal CT | Serum total tau was not strongly associated with CT findings in patients with mTBI | 4 |
| (55) | Su (2014) | Cross-sectional | 258 | Adult | Age range: 18-60 | CRP | PCS | Elevated baselines CRP levels were associated with a significant increase in the incidence of persistent PCS, persistent psychological problems and persistent cognitive impairment. | 6 |
| (56) | Svingos (2019) | Cross-sectional | 27 | Adult | No age range. Mean (SD): 18.8 (0.8) | miRNA | Symptom severity and recovery duration | No significant association between changes in miRNA expression and clinical test scores, acute symptom severity, or clinical recovery time. | 5 |
| (57) | Xu (2014) | Cross-sectional | 118 | Adult | Age range: 18-65 | Ghrelin | Cognitive deterioration | Decreased plasma ghrelin level was an independent predictor for three-month cognitive deterioration after mTBI. | 5 |
| (58) | Ye (2019) | Case-control | mTBI: 52, Control: 47 | Adult | No age range. mTBI Mean (SD): 34.48 (13.3). Control Mean (SD): 35.4 (12.0) | Alpha-synuclein | PPCS | Patients with lower alpha-synuclein presented more complaints on post-concussion symptoms and depression. | 5 |

## Secondary Outcomes Described in Included Studies

| **Secondary Outcome** | **Acronym or Notes** |
| --- | --- |
| Abnormal CT | - |
| Acute post-traumatic headache | APTH |
| Acute psychological symptoms | - |
| Brief Symptom Inventory Global Severity Index | BSI-GSI |
| Cerebral blood flow | CBF |
| Chronic mTBI | Having had at least 2 past mTBIs but none for at least 3 months |
| Chronic post-traumatic headache | CPTH |
| Clinical assessment scores | Including cognitive, balance and oculomotor testing |
| Cognitive function | - |
| Depressive symptoms | - |
| Fatigue | - |
| Glasgow Coma Scale | GCS |
| Glasgow Outcome Scale Extended | GOSE |
| Global neuronal connectivity | Gconn |
| Loss of consciousness | LOC |
| Number of symptoms | - |
| Persisting Symptoms Post Concussion | PPCS |
| Post-concussion syndrome | PCS |
| Post-traumatic amnesia | PTA |
| Post-traumatic headache | PTH |
| Recovery duration | - |
| Return to play | RTP |
| Return to work or school | RTWS |
| Sports related concussion | SRC |
| Symptom burden | Number of symptoms + symptom severity |
| Symptom clusters | - |
| Symptom severity | - |

## List of Definitions from Included Studies

| **Author** | **Definition** | **Abbreviation** |
| --- | --- | --- |
| American Congress of Rehabilitation Medicine | A blow to the head or acceleration/deceleration movement of the head resulting in one or more of the following: LOC <30 minutes, amnesia <24 hours or any alteration in mental state at the time of the injury. mTBI was defined as TBI cases with a Glasgow Coma Scale of 13-15. | American Congress of Rehabilitation Medicine |
| Centers for Disease Control and Prevention | A blow to the head or rapid acceleration/deceleration resulting in at least one of the following: loss of consciousness </= 30 minutes, post-traumatic amnesia </= 24h, neuropsychological abnormality with a Glasgow Coma Scale of 13 or greater within 30 minutes of injury. | Centers for Disease Control and Prevention |
| Centers for Disease Control and Prevention HEADS UP Educational Initiative | Sports related concussion is an injury resulting from a forceful bump, blow or jolt to the head that results in rapid movement of the head and causes a change in the athlete’s behaviour, thinking, physical functioning, or the following symptoms: headache, nausea, vomiting, dizziness/balance problems, fatigue, difficulty sleeping, drowsiness, sensitivity to light/noise, blurred vision, memory difficulty, and difficulty concentrating. | Centers for Disease Control and Prevention HEADS UP Educational Initiative |
| Consensus statement on concussion in sport - the 5th International Conference on Concussion In Sport 2016 | A concussion is caused by either direct or indirect forces acting on the head or neck resulting in short-term neurological impairment. | Consensus statement on concussion in sport 2016 |
| Department of Defence Evidence-based guidelines | Observed or documented alterations of consciousness and/or mental state (within 24 hours), less than 30 minutes of loss of consciousness, and/or post-traumatic amnesia lasting for as long as 1 day. | DoD evidence-based guidelines |
| European Federation of Neurological Societies | Patients presenting with a Glasgow Coma Scale score of 13-15 with loss of consciousness <30 minutes and post-traumatic amnesia <1 hour. | European Federation of Neurological Societies |
| Mild traumatic brain injury committee 1993 | Head trauma resulting in either memory loss or altered neurological function up to a day following the traumatic ictus or loss of consciousness for under 30 minutes. | - |
| Mild Traumatic Brain Injury Committee of the American Congress of Rehabilitation Medicine | A blow to the head or acceleration/deceleration of movement of the head resulting in one or more of the following: loss of consciousness (LOC) <30 minutes, amnesia <24 hours or any alternation in mental state and a Glasgow Coma Scale of >/= 13 measured 30 minutes more after injury. | MTBI Committee of the American Congress of Rehabilitation Medicine |
| National Collegiate Athletic Association Clinical Criteria | A traumatically induced physiological disruption of brain function, as manifested by at least one of the following: any loss of consciousness, any loss of memory for events immediately before or after the accident, any alteration in mental state at the time of the accident and focal neurological deficit(s) that may or may not be transient, but where the severity of the injury does not exceed the following: loss of consciousness of approximately 30 minutes or less. After 30 minutes, an initial GCS score of 13-15 and, post-traumatic amnesia not greater than 24 hours. | NCAA |
| National Institute for Health and Care Excellence Criteria | See full clinical guidelines (59) | NICE Criteria |
| Sports Concussions Assessment Tool 2 | An injury witnessed by an on-field certified athletic trainer and meeting the definition of concussion as defined by the Sport Concussion Assessment Tool 2. | SCAT2 |
| World Health Organisation Collaboration Centre for Neurotrauma Task Force | GCS of 13-15, loss of consciousness (LOC) for less than 30 min, post-traumatic amnesia (PTA) for less than 24 hours, with normal CT scan | WHO Collaboration Centre for Neurotrauma Task Force |
